# Geochemical and remote sensing insights into the hydrothermal origin of Banded Iron Formations at the Fatira Site, Northern Egyptian Nubian Shield

**DOI:** 10.1038/s41598-026-61319-7

**Published:** 2026-07-16

**Authors:** ElSayed A. Saber, Mohamed A. Abd El-Wahed, Omar R. Shalaby, Ahmed A. El-Sheikh, Mohamed Attia

**Affiliations:** 1 Geology Department, Faculty of Science, Sohag University, Sohag, 82524 Egypt; 2 Geology Department, Faculty of Science, Tanta University, P.O. Box: 31527, Tanta, Egypt; 3 State Key Laboratory of Continental Dynamics, Department of Geology, Northwest University, Xi’an, 710069 China; 4 Geology Department, Faculty of Science, Kafr El Sheikh University, P.O. Box: 33511, Kafr El Sheikh, Egypt

**Keywords:** Banded Iron Formation, Wadi Fatira, Egyptian Nubian Shield, Fatira Shear Zone, Hydrothermal solution, Sheared metavolcanic, Remote sensing, Planetary science, Solid Earth sciences

## Abstract

This study combines remote sensing, geochemical data, mineralogical analyses, and field investigations to examine the hydrothermal origin of the Banded Iron Formations (BIFs) in the Fatira region of the Egyptian Nubian Shield. The BIFs are situated within sheared metavolcanic sheeted dykes that form part of the ophiolitic mélange belt. These rocks show varying degrees of shearing due to the Fatira Shear Zone, which mainly consists of protomylonite, mylonite, and ultramylonite. The area is included in the Barud Gneissic Complex, along the ENE-trending dextral shear zone of the Qena-Safaga Line. The protolith underwent crustal shortening, leading to dextral movement along the Fatira Shear Zone. A study utilizing Landsat-8 imagery and the SAM method applied to ASTER data identified iron oxides, along with zones of chlorite and CO3-OH-bearing minerals, in the study area. The SAM algorithm mapped the distribution of common iron minerals, showing moderate to high lineament density and metavolcanic composition. The BIFs appear as thick single bands (3–4 m) along the outer edges of the shear zone and as thinner multiple bands within its central part. Field observations and mineralogical analyses indicate that the single bands consist of martitized magnetite-silicate facies, suggesting they are relatively unaltered. In contrast, the multiple bands comprise magnetite-chert-jasper facies, indicating significant alteration. Geochemical analyses support a hydrothermal genesis for the BIFs, with shearing planes acting as pathways for fluid flow. It is inferred that the protoliths of the BIFs originate from the host sheared metavolcanic sheeted dykes, which were affected by hydrothermal solutions. This research highlights the importance of integrating fieldwork, remote sensing, and laboratory analyses to understand better the formation and distribution of BIFs in tectonically active regions. The findings shed light on hydrothermal-related BIF formation within the sheared metavolcanic setting of the Wadi Fatira area and provide a useful framework for understanding iron mineralization in similar tectonically controlled environments.

## Introduction

Banded Iron Formations (BIFs) are chemical sedimentary rocks that precipitate in marine environments and typically contain around 15% iron^1,2^. These formations were deposited intermittently throughout the Archean and much of the Proterozoic, with peak deposition occurring around 2.5 billion years ago^3–5^. Classical low- to intermediate-grade metamorphic iron formations in weakly deformed basins have been extensively described in the literature, providing detailed insights into their petrographic, sedimentological, and geochemical characteristics^6^. These studies typically report microbandded to granular textures and relatively well-preserved sedimentary features in settings with limited tectonic deformation and hydrothermal influence. The formation of BIFs is often associated with hydrothermal processes, where iron-rich fluids interact with seawater to precipitate iron oxides and silicates^7–10^. Due to their economic potential, BIFs are considered the most profitable sources of iron. They have drawn significant attention from geologists, accounting for over 90% of the world’s exploitable iron resources and mineable iron ores^11,12^. Despite their economic importance, exploring and characterizing BIFs in tectonically complex regions, such as the Arabian-Nubian Shield, remains challenging. Traditional field-based methods are often time-consuming, costly, and cannot cover large and inaccessible areas^10,11,13^. Moreover, the geological processes responsible for forming and altering BIFs in these regions are not fully understood, particularly in areas affected by intense shearing and hydrothermal activity. This lack of understanding hinders the effective exploration and exploitation of iron resources in similar settings worldwide.

**Fig. 1:**
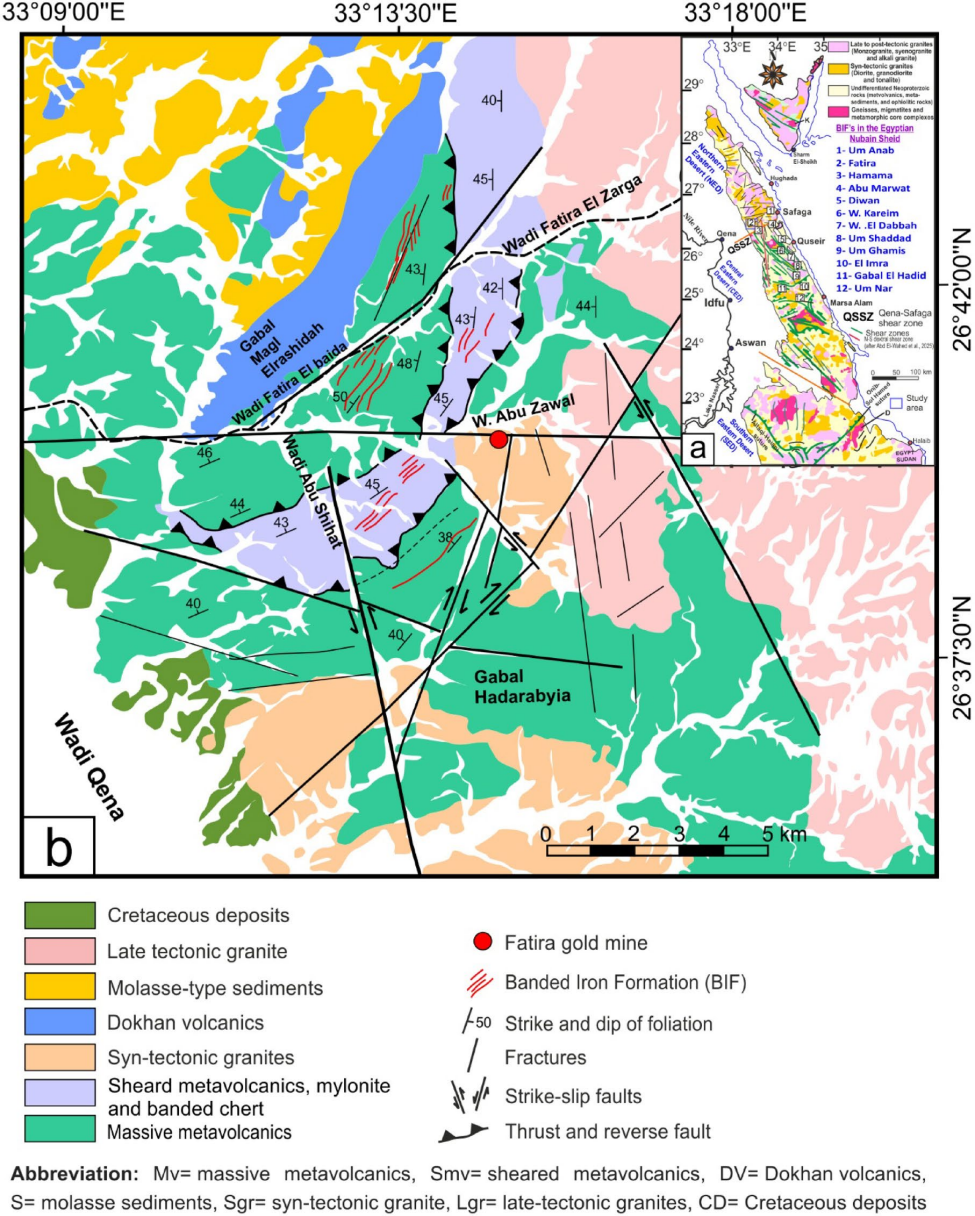
(**a**) Simplified geological map of the Eastern Desert of Egypt and Sinai showing the Najd fault zone and major structures in the Egyptian Nubian Shield, showing the location of the Banded Iron Formation. (modified from Abd El-Wahed and Attia^16^; Johnson et al.^25^; Zoheir et al.^26^), (**b**) Geological map of the Wadi Fatira area (modified and compiled after Conoco^27^; EGSMA^28^. These figures were created and processed by ENVI v. 5.6.2. software: https://www.l3harrisgeospatial.com/Software-Technology/ENVI), mainly used for image processing, and ArcGIS Desktop 10.8. (https://www.esri.com/en-us/arcgis/products/arcgis-desktop/overview/).

The Egyptian Nubian Shield (Fig. 1a) is located in northeastern Africa, spanning roughly 100,000 square kilometers and encompassing the Red Sea highlands within the Eastern Desert and southern Sinai^14^. This region consists of a variety of lithological elements, such as ophiolite slices, gneiss complexes, island arc metavolcanics, volcanoclastic metasediments, syn-tectonic granitoids, metagabbro, continental volcanics, molasse-type sediments, and late to post-tectonic granitoids^15–23^. The basement complex of the ENS is made up of gneiss complexes, amphibolites, and migmatites, which have been overthrust by a “suprastructural layer” consisting of low-grade imbricated ophiolitic sheets or mélanges, metavolcanics, and volcanoclastics^14^. The lithotectonic units have undergone tectonic amalgamation via thrusting during the accretion phase and have experienced approximately 600 million years of sinistral shearing along the northwest-directed shear zones of the Najd Fault System^18,19,23,24^.

More than 13 occurrences have been reported^11,29–31^. Additionally, minor occurrences have been reported in southern Sinai^32^and the western Gabal Kamel area in the southwestern desert of Egypt^33,34^. The ore reserves are estimated at 53 million tonnes^35^.

The BIF occurrences in the Fatira area (Fig. 1b) exist along the boundary between the Central and Northern Eastern Deserts of Egypt^7,36–38^. Due to insufficient aerial extension and economic potential, the Fatira BIF lacks the characteristics to qualify as a proper BIF ore type. Accordingly, this study focuses on the Wadi Fatira area in the Central Eastern Desert of Egypt, where BIF occurrences have been reported but remain poorly characterized.

Remote sensing techniques have gained popularity in mineral exploration due to their efficiency in saving time and effort compared to manual field surveys. They are particularly effective for lithological, structural, and hydrothermal mapping, thanks to the availability of diverse data sources^18,19,24,39–43^. Various spectral remote sensing methods, including image transformations, band mathematics, and algorithmic spectral techniques, have been applied both globally^44,45^ and locally in the Egyptian Eastern Desert for iron ore exploration^19,22,46–49^. To enhance geological and structural mapping, identify tectonic evolution, and determine the spatial distribution of iron ores in the area, we have integrated multisensor data, including Landsat-8, ASTER, Sentinel-1, and Digital Elevation Models (DEM), along with field data, petrographic investigations, and whole-rock chemistry analyses. This comprehensive approach aims to achieve our exploration objectives effectively.

By integrating field observations, remote sensing, mineralogical analyses, and geochemical data, this research aims to: (i) use advanced remote sensing techniques, identify the distribution and aerial extent of BIFs in the Wadi Fatira area, (ii) Describe the mineralogical and geochemical characteristics of the Banded Iron Formations (BIFs) and their associated host rocks to comprehend the geological processes that led to their formation, and (iii) Evaluate the role of hydrothermal activity in the alteration and enrichment of iron deposits in tectonically active regions.

The findings of this study have broader implications for exploring BIFs in similar geological settings worldwide. This research demonstrates the effectiveness of integrated remote sensing, field observations, and mineralogical and geochemical analyses. It provides a cost-effective, efficient approach to mineral exploration in remote, inaccessible areas. Furthermore, insights into the hydrothermal processes responsible for BIF formation can inform future exploration strategies and support the sustainable exploitation of iron resources. This study also advances our understanding of the geological evolution of the Arabian-Nubian Shield, offering new perspectives on the tectonic and hydrothermal processes that shaped this region during the Neoproterozoic.

## Geological setting

**Fig. 2:**
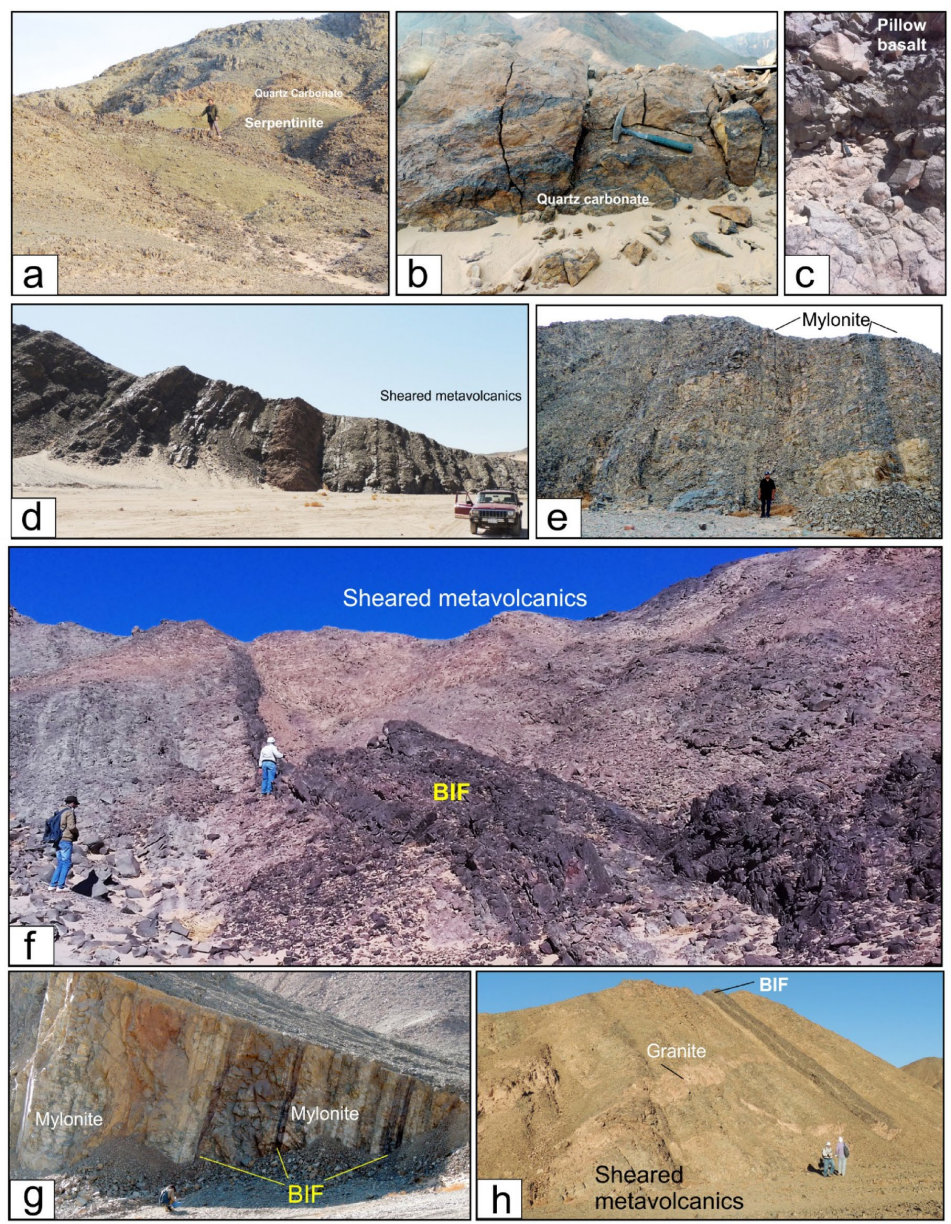
(**a**) Sheared metavolcanics (mylonite) with serpentinites pockets and quartz carbonate (**b**) Band of quartz carbonate, (**c**) Pillow basalt in massive metavolcanics, (**d**) Subvertical metavolcanic sheeted dykes and their sheared derivatives intruded by a felsic dyke, (**e**) Sheared metavolcanics exhibiting subvertical mylonitic bands, (**f**) Field photograph showing isolated BIF bands; note relict remnants of mylonite preserved within the BIF, (**g**) Banded Iron Formation separated by a thin ultramylonite bands, (**h**) Sheared metavolcanics hosting single-occurring BIF bands and intruded by monzogranite.

The Wadi Fatira area (Fig. 1b) forms part of a Neoproterozoic ophiolitic mélange that comprises lenticular blocks of serpentinite (Fig. 2a) with quartz carbonate (Fig. 2b), pillow basalt (Fig. 2c), and metavolcanic (Fig. 2d,e). The mélange is structurally associated with syn-tectonic granitoids, Dokhan volcanics, molasse-type sediments, and late-orogenic granitoids^50–52^. The area features felsic, intermediate, and basic dikes, representing the most recent magmatic products that intersect earlier lithologies and are influenced by numerous faults and joints throughout the study area.

In the field, the dismembered ophiolite is exposed as thin sheets of talc-carbonate rocks, interspersed with lenticular pockets of serpentinite and quartz-carbonate ridges (Fig. 2a,b). These lithologies are embedded within a matrix dominated by talc schist and quartz-carbonate mylonites. The serpentinite occurs as small, isolated relics and pockets, typically displaying pale grey to pale green hues. The talc-carbonate sheets are characterized by their cream to brown coloration and a distinctly cavernous texture. Quartz-carbonate rocks appear as reddish-brown ridges and are medium- to fine-grained in texture (Fig. 2b).

The Fatira Shear Zone significantly affects the metavolcanic rocks, predominantly of intermediate to mafic composition (e.g., meta-andesite and meta-basalt). This major tectonic feature has caused variable degrees of shearing, resulting in intensely deformed metavolcanic sheeted dykes^52^. These rocks occasionally encase thin layers of metamorphic banded iron formation (BIF) found at Gabal Magl Elrashayda.

The metavolcanic and their sheared derivatives (Fig. 1b) are penetrated by a series of granodiorite batholiths situated along shear zones. The shearing process is distinct from the granite intrusion. It is marked by mylonitic, significantly altered grey granite, along with the intergrowth of hornfelsic rocks, resulting from contact metamorphism and intense deformation due to the granitic intrusion^51^. The metavolcanic rocks are found as vertical to steeply inclined, stacked, greyish-green to dark-green sheeted dykes (Fig. 2d,e). The sheared metavolcanics span an area of approximately 6 km² in the central region of the Fatira Shear Zone and display a spectrum of shear intensities, ranging from protomylonites to banded ultramylonites^53^ (Fig. 2e), along with remnant sigmoidal lenses of metadolerite and metabasalt.

In the Wadi Fatira region, the BIFs are situated within highly sheared metavolcanic sheeted dykes, predominantly made up of protomylonite, mylonite, and ultramylonite (Fig. 2f,g). The shearing of these rocks is influenced by the Fatira Shear Zone, which serves as a conduit for hydrothermal fluids, facilitating the formation and modification of the BIFs. The concentration of BIFs along the boundaries of the shear zone and within its central region indicates a significant tectonic influence on their spatial distribution and mineralization.

Syn-tectonic granitoids (Fig. 1b) consist of granodiorite and tonalite, forming a north-south elongated body intruded by late-to-post-granitoid rocks and various dike swarms with differing trends. This body exhibits significant jointing, fracturing, exfoliation, and gneissose structures and contains xenoliths of metavolcanic origin.

The Dokhan Volcanic rocks (Fig. 1b) are located in the northwestern region of the study area. They are characterized by rhyolite porphyry, forming northeast-elongated bodies with a reddish to massive pinkish hue. The molasse-type sediments (Fig. 1b) consist of polymictic conglomerates in the lower section, which transition into greywackes and siltstone. All rock units are intruded by granitoid masses (Fig. 2h), ranging from monzogranite to syenogranite. The syenogranite rocks constitute the core of the monzogranite block. The study area is intersected by various types of parallel and subparallel dike swarms, including felsic, intermediate, and mafic affinities (Fig. 2d). The acidic dikes are predominantly represented by NE-trending felsite dikes, quartz veins, and steeply dipping andesitic dykes.

## Materials and methods

### Multi-sensor remote sensing data and processing

A set of free-cloud terrain corrected scenes of remotely sensed datasets comprising optical data (e.g., Landsat-8 OLI and ASTER) acquired from the United States Geological Survey (USGS) and NASA Earth Data Center, radar data (Sentinel-1 A) obtained from https://search.asf.alaska.edu and Digital Elevation Model data (DEM) from NASA Earth Data Center have been processed using a package of software (e.g., Envi 5.3, ArcMap 10.8, PCIgeomatica 2016, RockWork2016, Coreldraw-64 bit) for the lithological discrimination, structural mapping, lineament extraction and allocation of iron oxides in the investigated area. The satellite data have been projected utilising the WGS-84 UTM Zone 36 N coordinate system. Moreover, they were stacked and subsetted to outline only the examined study area.

With eleven bands of Landsat-8 OLI\TIRS covering different spectral ranges and spatial attributes (Table 1, Roy et al.^54^) and bands for ASTER covering visible and near-infrared (VNIR), short-wave infrared (SWIR), and thermal infrared regions (TIRS) (Table 1; Abrams et al.^55^), the VNIR and SWIR bands of Landsat OLI (B2 to B7) and ASTER (B1 to B9) were employed to unravel the spatial distribution of iron oxide and identify the lithological units, besides the structural mapping of the area under consideration. Moreover, the dual-polarised Sentinel-1 A (Table 2) radar data have been used for lineament extraction alongside DEM imagery, which served as a base map for the surface distribution of each lithological unit, structural element, extracted lineament, and iron oxide minerals. The rationale for selecting specific spectral bands in this analysis is rooted in the principles of mineral reflectance spectroscopy. The targeted mineral assemblages, including iron oxides/hydroxides, hydroxyl-bearing minerals, and carbonates, exhibit diagnostic absorption features in the VNIR and SWIR regions. These features arise from two primary mechanisms: (1) electronic transitions in constituent ions such as Fe²⁺ and Fe³⁺, and (2) vibrational overtone and combination tones of fundamental molecular bonds (e.g., Al-OH, Mg-OH, CO₃)^56,57^. Therefore, the satellite bands were explicitly chosen to align with the known positions of these key absorptions. For instance, the ~ 2.1–2.4 μm SWIR region is critical for discriminating hydroxyl-bearing minerals based on the wavelength shift of their Al-OH, Fe-OH, and Mg-OH absorption features^58^. At the same time, specific VNIR bands are essential for mapping iron oxide phases via their distinct Fe³⁺ charge-transfer bands^56^. This targeted approach, supported by the canonical works^19,49,57,59–61^ ensures the analytical capability to detect and differentiate the mineralogical units of interest, including the iron oxides.

**Table 1:** The highlighted aspects of the optical data.

Landsat-8				ASTER			
Band (B)	Wavelength (µm)	Resolution (m)	Spectral region	Band (B)	Wavelength (µm)	Resolution (m)	Spectral region
B1	0.435–0.451	30	Coastal	B1	0.52–0.60	15	VNIR
B2	0.452–0.512		Blue	B2	0.63–0.69		
B3	0.533–0.590		Green	B3	0.76–0.86		
B4	0.636–0.673		Red	B4	1.60–1.70	30	SWIR
B5	0.851–0.879		NIR	B5	2.145–2.185		
B6	1.566–1.651		SWIR	B6	2.185–2.225		
B7	2.107–2.294			B7	2.235–2.285		
B8	0.503–0.676	15	Panchromatic	B8	2.295–2.365		
B9	1.363–1.384	30	Cirrus	B9	2.360–2.430		
B10	10.60–11.19	100	TIR	B10	8.125–8.475	90	TIR
B11	11.50–12.51			B11	8.475–8.825		
–	–			B12	8.925–9.275		
–	–			B13	10.25–10.95		
–	–			B14	10.95–11.65		

Near Infrared “NIR”, Short wave Infrared “SWIR”, Thermal Infrared “TIR”, Visible Nera Infrared “VNIR”.

**Table 2:** The highlighted aspects of the Sentinel-1 radar data.

AM	SM	IW	EW	WV
Beam mode	S1 to S6	IW1 to IW3	EW1to EW5	WV1&WV2
Center frequency	C-band (5.405 GHz)			
Polarization	SP (HH or VV) DP (HH + HV and VV + VH)			
Spatial resolution (range x azimuth) (m)	5 × 5	5 × 20	25 × 100	5 × 20
Band width (Km)	80	250	4	20 × 20
Chirp bandwidth (MHz)	87.6–42.2	56.5–42.8	22.2–10.4	74.5 & 48.2
Incidence angle (deg)	20–43°	30–42°	20–44°	23 & 36.5°

Acquisition mode “AM”, Stripmap “SM”, Interferometric wide swath “IW”, Extra wide swath “EW”, Wave “WV”, Single Polarization “SP”, Daul Polarization “DP”, Degree “deg”.

The multisensory data was processed through two stages: pre-processing and processing. For pre-processing, the Internal Average Relative Reflection (IARR), a type of atmospheric correction, was applied to Landsat-8 and ASTER data to mitigate or eliminate atmospheric influences; this could potentially affect the quality of outcomes from the processing phases (e.g., image classification and mineral identification). To reduce speckle in Sentinel-1 A (S1A) radar imagery, the enhanced Lee filter was applied across both polarizations (VH and VV), preserving texture information. At the same time, the DEM was processed using spatial analysis tools in ArcMap.

The image transformations including False Color Composite (FCC), Decorrelation, Band Ratio (BR), stretch and Principal Component Analysis (PCA) representing the processing stage, have been applied on the optical data to demarcate the lithological units, trace the major structure features and mapping the iron ores in the presented area alongside the a spectrum matching technique “Spectral Angle Mapper” (SAM) which applied on the first nine bands of ASTER data to monitor the spatial distribution of iron oxides including hematite, magnetite, goethite and Jarosite. The Spectral Angle Mapper monitoring the presence of wadis or lineaments, such as fractures, joints, and faults. At the same time, decorrelation stretching enhances color diversity within a color image, improving clarity and reducing redundancy. Band rationing is a crucial technique for enhancing images, which can be executed by dividing the pixel values of one band by those of another or by employing various mathematical operations. PCA is a mathematical approach that produces uncorrelated output bands, effectively isolates noise components, and diminishes redundant information across different bands through a rotational method. PCA is a practical approach for generating multispectral images that aid geological interpretation and mineral detection.

The selection of the Spectral Angle Mapper (SAM) algorithm for this study was based on its proven efficacy in mineralogical mapping, particularly with ASTER data. SAM determines spectral similarity by calculating the angle between image pixel spectra and reference endmember spectra in a multi-dimensional space, focusing on spectral shape to reduce the effects of illumination and albedo variations. For mapping iron oxides and associated minerals, the analysis used the first nine ASTER bands, covering both the Visible and Near-Infrared (VNIR) and the Shortwave Infrared (SWIR) regions. The VNIR bands are crucial, as iron oxides such as hematite and goethite exhibit diagnostic features there due to electronic processes involving Fe³⁺ ions. Concurrently, the SWIR bands are sensitive to vibrational processes in molecules, enabling the detection of hydroxyl-bearing and sulfate minerals such as jarosite. This integrated VNIR-SWIR approach allows for more robust and discriminatory mapping of mineral assemblages by leveraging the distinct physical processes governing spectral reflectance in each region, a methodology successfully demonstrated in practical case studies for extracting alteration minerals^19,62,63^.

Furthermore, band math was applied on the S1A to generate the VH + VV composite, which was subsequently combined with the two polarizations, VH and VV, into a singular layer processed by PCA technique to yield the three principal components (PC1, PC2, PC3) for the automatic extraction of the linear structures. All geospatial figures were generated and processed using ENVI v.5.6.2 (L3Harris Geospatial), which was employed for its advanced image processing capabilities, and ArcGIS Desktop 10.8 (Esri Inc.), which was utilized for spatial analysis and cartographic presentation.

### Field studies

Fieldwork in the Wadi Fatira region was carried out over three field trips to examine the Banded Iron Formation (BIF) and its associated host rocks. The investigation included systematic traverse mapping and thorough outcrop sampling, informed by an existing geological map (1:50,000) and remote sensing data. Thirty-five rock samples were gathered, comprising 15 from the BIF, 15 from the host metavolcanic rocks (massive and sheared), and five from hydrothermal alteration zones (such as banded chert). Field observations encompassing lithology, structural characteristics, and mineralogical differences were recorded with photographs and GPS coordinates. The fieldwork was complemented by remote sensing analysis to confirm spectral signatures and guarantee focused sampling of iron-rich areas.

### Laboratory work

Petrographic and mineralogical studies were conducted on 25 thin-sections of the Banded Iron Formations (BIFs) and their associated host rocks, using a reflected-transmitted polarizing microscope. These thin-polished sections were prepared in the mineral and rock preparation laboratory in the Geology Department at Assuit University. The major oxide compositions (wt%) and trace element concentrations (ppm) were analyzed for 10 samples from the Banded Iron Formation, 10 samples from massive metavolcanics, 10 samples from sheared metavolcanics, and five samples from banded chert, employing a Philips X-ray fluorescence technique (model PW/2404) equipped with a Rh radiation tube. The detection limits for major oxide compositions range from 0.001 to 0.03%, while those for trace elements vary from 0.01 to 0.5 ppm. The loss on ignition (L.O.I.) for the samples was assessed by heating 0.5 g of the powdered sample at 1000 °C overnight. The Rare Earth Element (REE) concentrations for 11 samples from the BIF and host rocks were determined using inductively coupled plasma mass spectrometry (ICP-MS) (720 ICP-OES, Agilent Technologies), which has detection limits of 0.1 ppm. The geochemical analyses were conducted at the central laboratories of the Egyptian Mineral Resources Authority (EMRA), Egypt.

## Results

### Remote sensing

#### Lithological demarcation

**Fig. 3:**
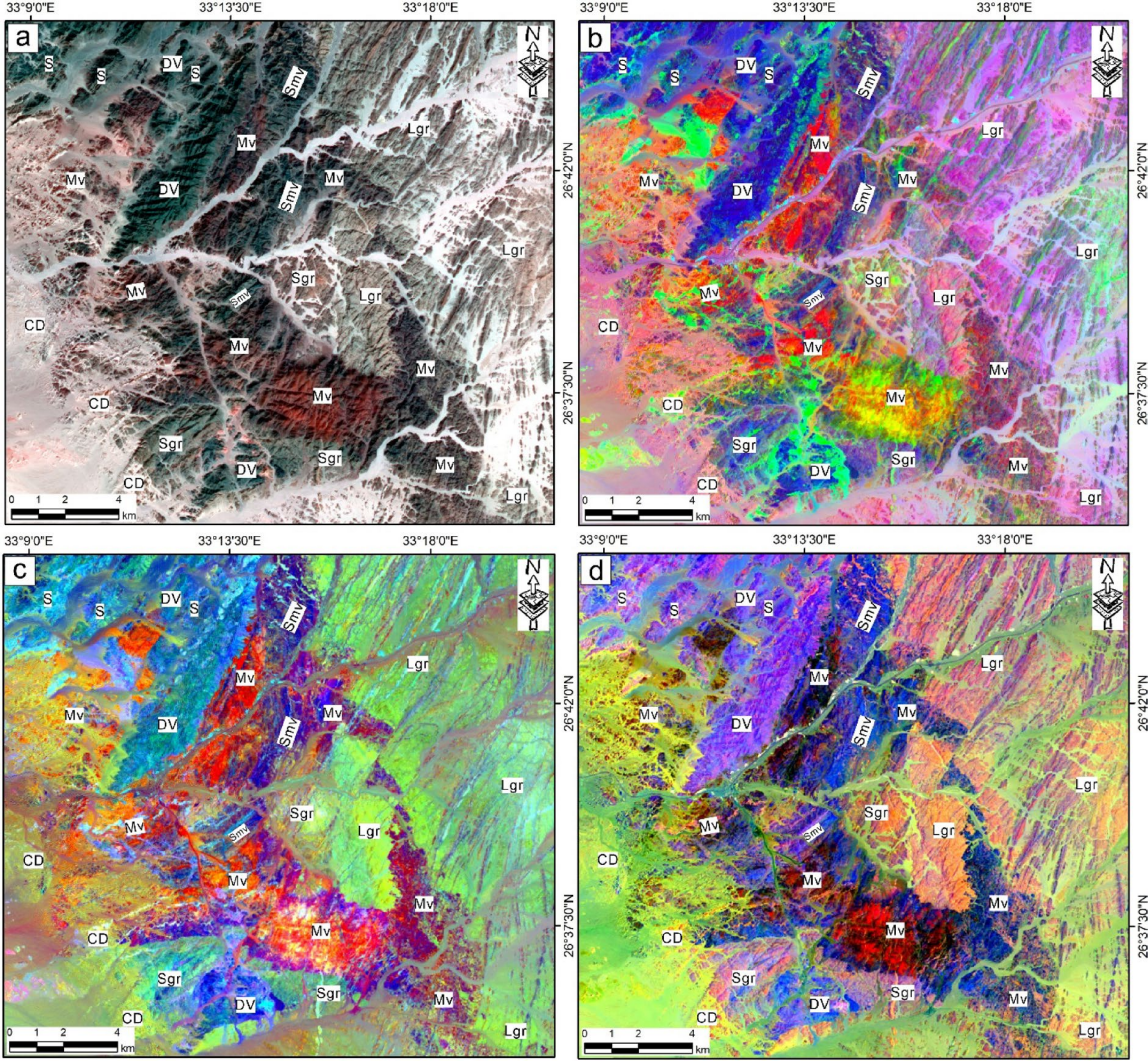
Lithological discrimination utilizing; (**a**) FCC 432-RGB of ASTER, (**b**) Decorrelated FCC 765-RGB, (**c**) Kaufmann ratio 7/5 5/4 6/7-RGB and (**d**) Br 4/5 4/2 5/7-RGB of Landsat-8. Mv=massive metavolcanics, Smv=sheared metavolcanics, DV=Dokhan volcanics, S=molasse sediments, Sgr = syn-tectonic granite, Lgr=late-tectonic granites, CD= Cretaceous deposits. This figure was created and processed by ENVI v. 5.6.2. software: https://www.l3harrisgeospatial.com/Software-Technology/ENVI), mainly used for image processing, and ArcGIS Desktop 10.8. (https://www.esri.com/en-us/arcgis/products/arcgis-desktop/overview/).

The tonal variations of the chosen color composites, including ASTER FCC 432-RGB, decorrelated FCC 765, Kaufmann ratios 7/5, 5/4, and 6/7, and Br 4/5, 4/2, and 5/7 in Landsat-8 RGB, helped discriminate among the lithological units and trace the different valleys in the presented area. Due to the higher spatial resolution of ASTER (15 m), the FCC 432-RGB was employed mainly for drawing the primary and secondary valleys alongside the distinguishing of the majority of rock units by unique colors, for instance, bloody brown to brownish red for massive metavolcanics, ocean green for sheared metavolcanics, moss green for Dokhan volcanics and molasse sediments, pale blue-green to pale orange-brown for syn-tectonic granites, light brown for the late-tectonic granites, and reddish white for Cretaceous sediments (Fig. 3a).

Decorrelated FCC 765-RGB (Fig. 3b) successfully highlighted the lithological borders among the rock units. It marked the massive metavolcanics in red to yellowish red, sheared metavolcanics in reddish blue, Dokhan volcanics in deep blue, molasse sediments in greenish deep blue, syn-granites in greenish violet to bluish violet, late-granites in purple, and Cretaceous sediments in pale orange-pink. In the Kaufmann ratio 7/5 5/4 6/7-RGB, the massive metavolcanics appear in deep orange to greenish orange. In contrast, sheared metavolcanics are deep blue to reddish-deep blue, which are considered the source of BIF in the presented area (Fig. 3c). Moreover, the Dokhan volcanics are distinguished by green-blue tones. In contrast, the molasse sediments appear in cyan tones, and the late granitic features are characterized by lemon color. The differentiation between the two granitic types was apparent using Br 4/5 4/2 5/7-RGB (Fig. 3d), where the syn-granites were recognized by pale pinkish-violet pixels. At the same time, the late-granites appeared in pure pale orange to pinkish orange pixels. The massive metavolcanics are also distinguished by red hues from the other units (Fig. 3d).

#### Iron oxide monitoring

**Fig. 4:**
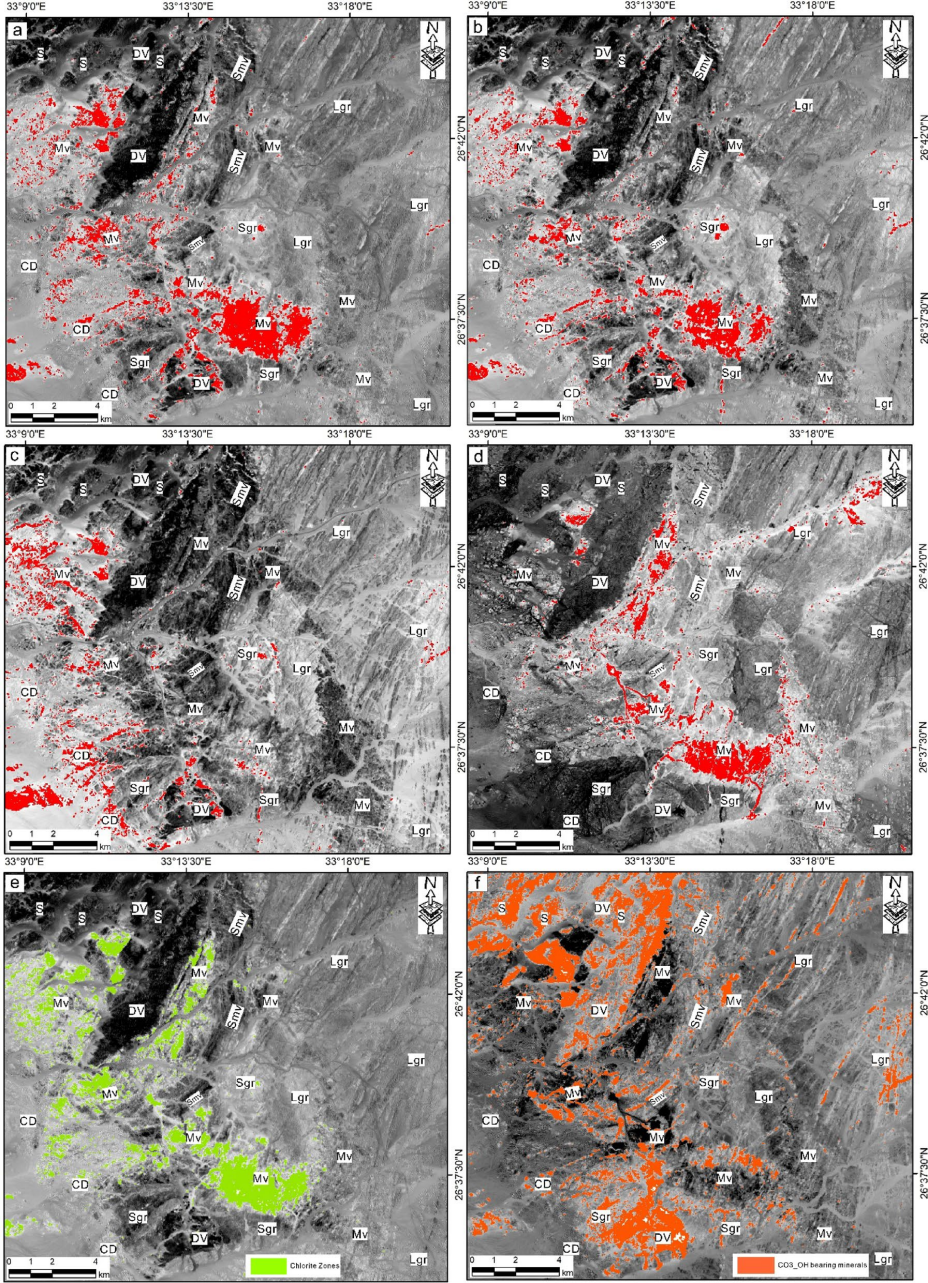
Iron and alteration zones monitoring via grey scale imageries of Landsat-8; (**a**) 6/2, (**b**) 6/4 and (**c**) 6/5 band ratios, (**d**) PC 3, (**e**) BR 7/5 for chlorite zones; (**e**) BR 6/7 for CO_3_-OH bearing minerals zones. See Fig. 1 for abbreviations. This figure was created and processed by ENVI v. 5.6.2. software: https://www.l3harrisgeospatial.com/Software-Technology/ENVI), mainly used for image processing, and ArcGIS Desktop 10.8. (https://www.esri.com/en-us/arcgis/products/arcgis-desktop/overview/).

To delineate occurrences of iron ore minerals, various grey-scale band ratios from Landsat-8, along with the SAM method applied to ASTER data, were used to detect iron oxides in the study area. The grey scale BRs of Landsat-8 include 6/2, 6/4, and 6/5 as well as PC3, which were utilized as indicative ratios for the occurrence of ferrous and ferric iron oxides (Fig. 4a–d). The detected iron oxide areas have been colored as red patches using ROI tools in Envi-64-bit to distinguish them from the surrounding areas. Moreover, the grey-scaled ratios 7/5 and 6/7 (Fig. 4e,f) were employed to allocate the chlorite- and CO_3_-OH-bearing mineral zones, respectively, which are considered standard alteration zones where hydrothermal fluids can react with the country rocks, allowing the precipitation of some iron oxides.

**Fig. 5:**
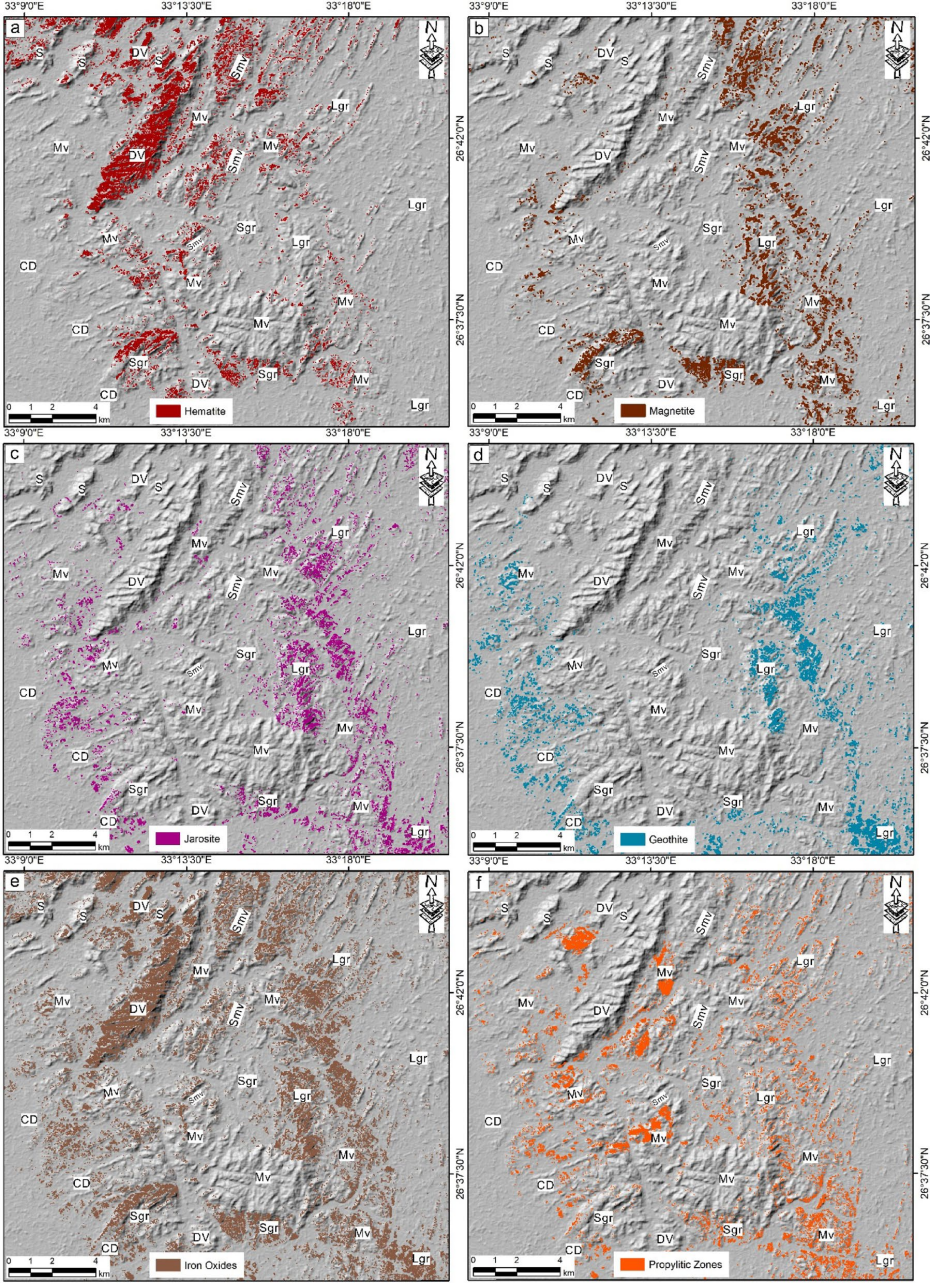
Iron and alteration zones monitoring via SAM grey scale imageries of ASTER; (**a**) Hematite, (**b**) Magnetite, (**c**) Jarosite, (**d**) Goethite, (**e**) Spatial distribution of the merged iron oxides, (**f**) Propylitic zones. See Fig. 1 for abbreviations. This figure was created and processed by ENVI v. 5.6.2. software: https://www.l3harrisgeospatial.com/Software-Technology/ENVI), mainly used for image processing, and ArcGIS Desktop 10.8. (https://www.esri.com/en-us/arcgis/products/arcgis-desktop/overview/).

Based on the spectral signatures for the four iron oxide minerals (hematite, magnetite, Jarosite and goethite) sourced from the USGS spectral library supported by Envi-5.3, a series of processing steps were conducted on the nine ASTER bands using the SAM algorithmic method to allocate the spatial distribution of the four iron oxides by generating a collection of grayscale images, complemented with color visuals that highlight the most significant regions of the iron minerals (Fig. 5a–d). To create the SAM images of the four iron minerals, the rule threshold was set to the specific value shown in Table 3; at this point, each mineral began to emerge, covering different areas with varying percentages (Fig. 5a–d). Consequently, the four iron minerals have been merged as one item and dropped on the hill shade map to display the general surface distribution of the iron through the examined area (Fig. 5e). Furthermore, the propylitic zones which are characterized by chlorite, epidote, calcite and talc minerals were allocated using the SAM technique (Fig. 5f). These zones considered one of the probable areas where the hot fluids reacting with the country rocks allowing the deposition of iron ores.

**Table 3:** Static summary of the SAM technique.

Minerals/alteration zone	Mineral	Rule threshold	Method	Target count	Average area km^2^
Iron oxides	Hematite	0.500	SAM	3849	4089.9260
	Jarosite	0.900		3623	2905.7480
	Magnetite	0.450		2599	2050.9714
	Geothtie	0.700		2882	2423.6294
Propylitic	Calcite	0.800		2461	1645.8553
	Chlorite	0.800		3384	2466.7554
	Epidote	0.800		3380	2631.7678
	Talc	0.800		2181	1491.3342

#### Automatic lineament extraction via S1A

**Fig. 6:**
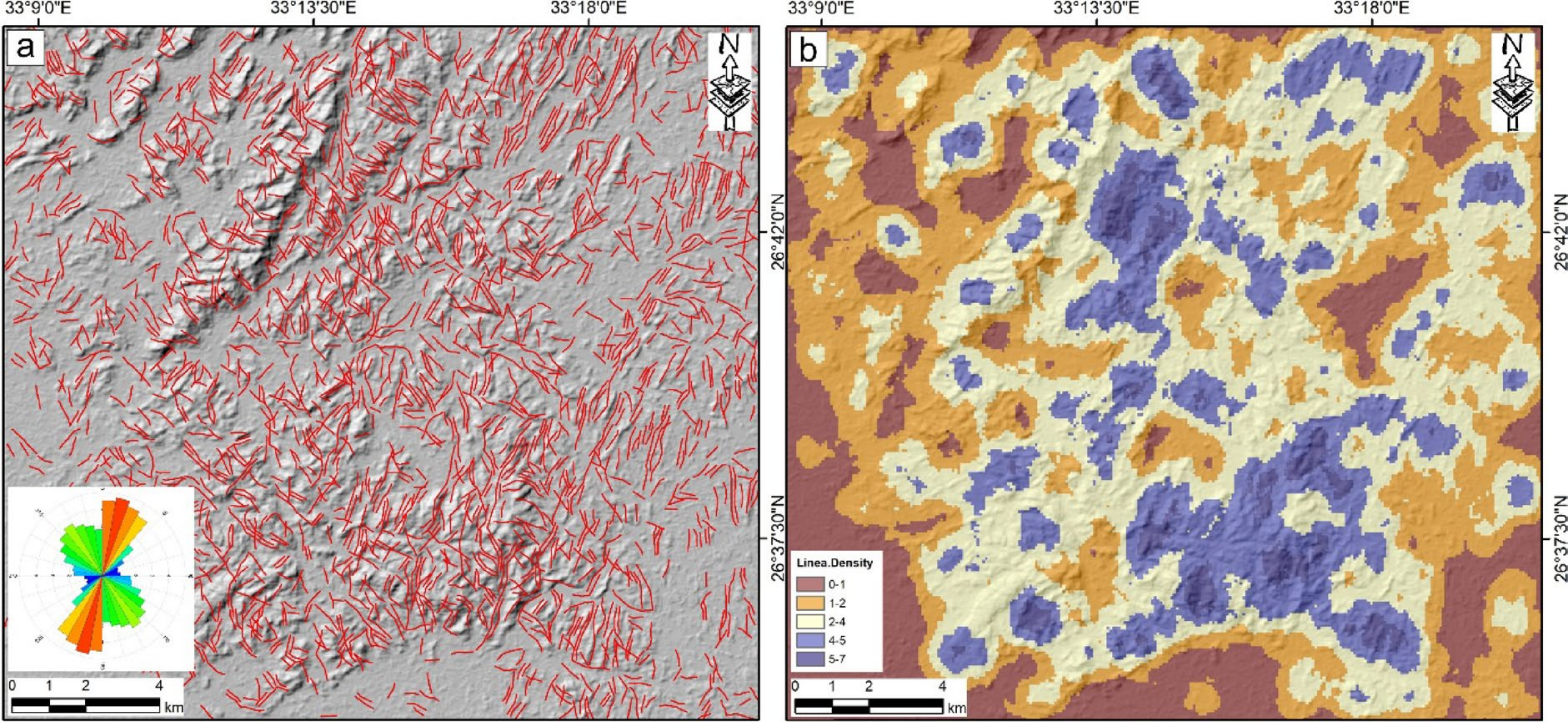
Automatic lineament extraction using S1A radar data; (**a**) Lineament map plus azimuth diagram, (**b**) Lineament density map. This figure was created and processed by ENVI v. 5.6.2. software: https://www.l3harrisgeospatial.com/Software-Technology/ENVI), mainly used for image processing, and ArcGIS Desktop 10.8. (https://www.esri.com/en-us/arcgis/products/arcgis-desktop/overview/).

As previously noted, a single layer of S1A data was processed using PCA, yielding three principal components (PC1, PC2, PC3). Since PC1 encompasses most information, it was selected for further processing to facilitate the automatic extraction of lineaments (Fig. 6a) and create a lineament density map (Fig. 6b). The extracted lineaments (Fig. 6a) were processed using RockWork16 to generate a frequency diagram that emphasizes the primary trends within the study area (Fig. 6a). Excluding regions covered by recent deposits, the extracted lineaments appeared as intense red, short lines that are distributed and interspersed throughout nearly all rock units (Fig. 6a).

Based on the azimuth diagram (Fig. 6a), the predominant orientations of lineaments are NE, NNE, NW, NNW, and E-W, listed in order of decreasing significance. The density map (measured in numbers per square kilometer; Fig. 6b) illustrates the distribution of linear segments across the entire region. This density map indicates that lineaments are highly to moderately concentrated over the metavolcanics and parts of the syn and late granites.

The deformation history of the Egyptian Nubian Shield includes five notable deformation events^19,23^. The first phase, which involves N-S shortening (D_1_), is characterized by the development of S1 regional foliation, thrusts dipping towards the N and NE, and east-west-trending folds. The second phase, marked by NE-SW shortening (D_2_), has resulted in NW-trending structures, including an S2 axial-planar foliation striking NW-SE, NW-trending folds (F_2_), and sinistral shear zones associated with the Najd Fault System. The third event, known as E-W shortening (D_3_), features N-S trending open folds (F_3_) and S3 axial planar foliation, along with dextral shearing (D_3_) occurring along the NW-trending shear zones (D_2_). The continuation of the E-W shortening event (D_4_) has produced NE-trending folds (F_4_), S_4_ axial planar foliation, dextral shearing along NW-trending shear zones, and the formation of NE-trending dextral shear zones. In the fifth phase (D_5_), the Egyptian Nubian Shield is impacted by brittle strike-slip faults oriented in ENE-WSW, NE-SW, and N-S directions. These faults formed during the Early Cretaceous period, coinciding with the rifting of the Red Sea. The subsequent brittle structures have led to displacements in thrust faults and fold axes.

The Wadi Fatira area constitutes a minor segment of the Barud Gneissic Complex, located along the ENE-trending dextral shear zone of the Qena–Safaga Line, which acts as a crucial tectonic boundary separating the basement terrains of the Northern and Central Eastern Desert. The protolith of the Barud Gneissic Complex underwent crustal shortening approximately 697 Ma in a NW–SE orientation, which initiated dextral movement along the Fatira Shear Zone^64^. Around 630 Ma, substantial granodiorite/tonalite complex batholiths intruded into the Barud Gneissic Complex protolith along the Qena–Safaga Line, occurring at relatively shallow crustal depths^64^.

The Fatira Shear Zone spans a significant area, with multiple curvilinear strike-slip faults extending over 70 km across the visible width of the Egyptian Nubian Shield. It gradually fans out towards the west and south, with its width increasing from a few kilometers in the east to several tens of kilometers in the west^64^. The northern and southern limits of this zone are marked by narrow zones of ultramylonite, phyllonite, and sheared metavolcanics. The Wadi Fatira area signifies the western end of the Fatira Shear Zone, where highly sheared metavolcanics and mylonite thrust over metavolcaniclastics, forming a transpressional imbricate thrust zone.

The Banded Iron Formations (BIFs) located in the Wadi Fatira region are found within extensively sheared metavolcanic sheeted dykes, which are predominantly made up of protomylonite, mylonite, and ultramylonite (Fig. 2f–h). The shearing of these rocks varies due to the influence of the Fatira Shear Zone, which serves as a conduit for hydrothermal solutions, facilitating the development and alteration of the BIFs. The concentration of the BIFs along the boundaries of the shear zone and within its central region indicates a significant tectonic influence on their distribution and mineralization.

**Fig. 7:**
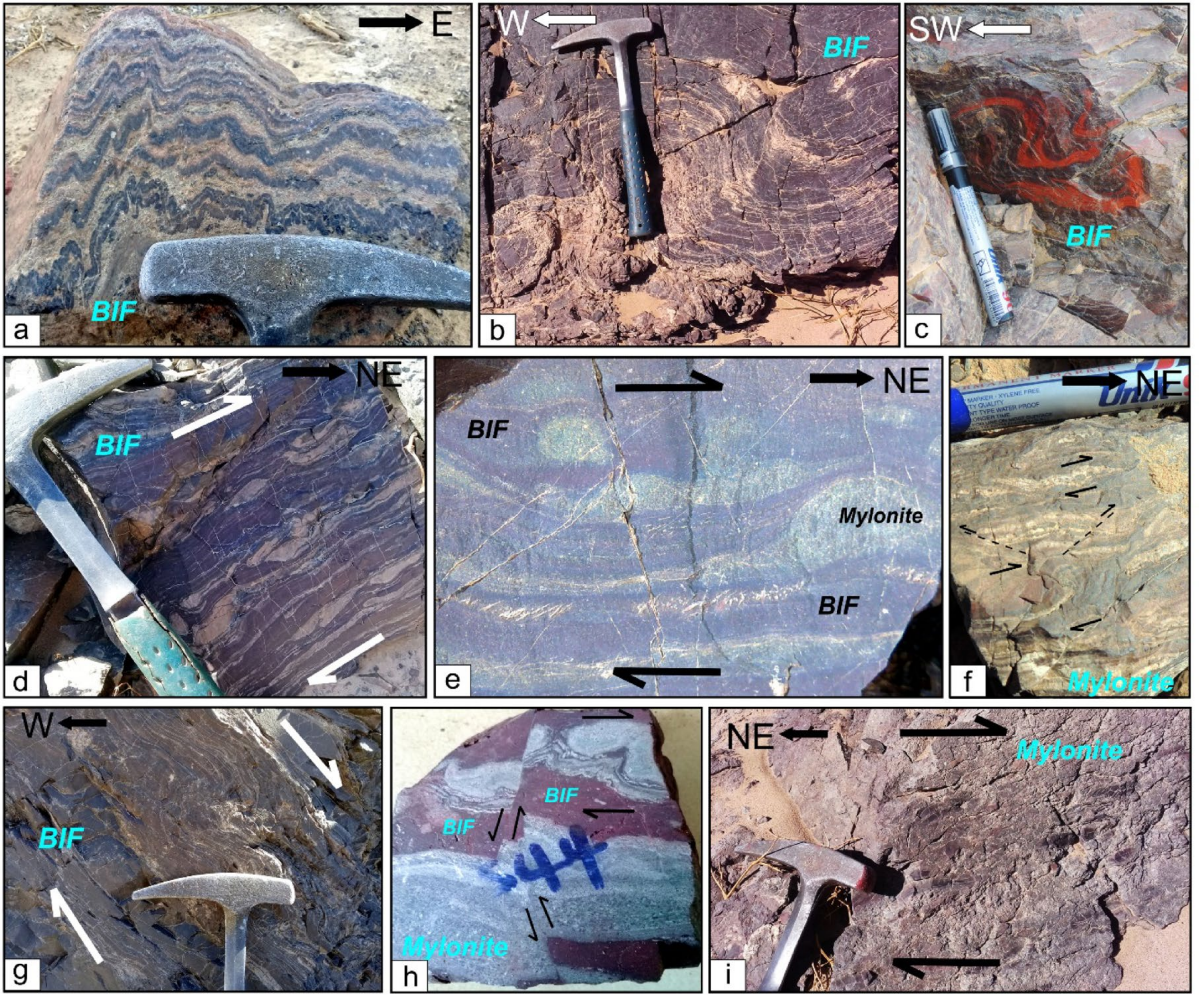
Fold structure in the BIF (**a**) refolded asymmetrical fold, (**b**) box fold, (**c**) overturned fold. Shear sense indicators suggesting dextral sense of shear (**d**) and (**e**) boudinaged mylonite clasts in BIF, (**f**) Small-scale pop-up in mylonite, (**g**) asymmetrical folds, and (**h**) sigmoidal porphyroclasts and lithic fragments of metabasalts and meta-andesite in mylonite.

The Fatira region exhibits two significant deformation phases, labelled D4 and D5^19,23^. The D_4_ event is characterized by schistosity and a stretching lineation within the Fatira El Beida sequence. The schistosity direction varies from N–S to N30°E, with a general dip towards the west at about 45°. Additionally, there are NNE-to NE-trending mesoscopic asymmetric, open, overturned, and box folds (Fig. 7a–c) with SE vergence, as well as NE-trending banding in the Banded Iron Formation (BIF) and sheared metavolcanics.

Mylonitic foliation in the Fatira Shear Zone is parallel to the bedding planes in the BIFs. During ductile deformation within a shear zone, mylonitization often exploits pre-existing planes of weakness, such as bedding planes in sedimentary rocks^53^. When the direction of tectonic shearing is sub-parallel to the original bedding, mylonitic foliation can develop parallel or nearly parallel to these bedding planes. In such cases, the resulting mylonitic foliation may overprint or be superimposed on the primary sedimentary bedding, and relics of the original bedding may be preserved within the mylonite^53^. Petrographic observations may reveal aligned recrystallized minerals, stretching lineations, and other microstructures indicative of mylonitization, with foliation parallel to bedding^53^.

Shear-sense indicators suggest a dextral shearing sense along the western part of the Fatria shear zone. These indicators include boudinaged mylonite clasts in BIF (Fig. 7d,e), small-scale pop-up in mylonite (Fig. 7f), asymmetrical folds (Fig. 7g,h), and sigmoidal porphyroclasts, lithic fragments of metabasalts and meta-andesite in mylonite (Fig. 7i). The D_5_ event is the most significant deformation affecting the study area, marked by major strike-slip shear zones that trend NW–SE with a moderate to steep dip towards the NE. Lithological offsets indicate a sinistral movement, and these faults disrupt all rock inputs and structures. The orientation of faults and joints is predominantly NE-SW, E-W, and NNE-SSW, with minor orientations of NW-SE and N-S.

Field observations further emphasized the influence of structural control within the shear zone on the distribution of Banded Iron Formations (BIF), with NE-SW-oriented shear zones serving as pathways for hydrothermal fluids that modified the metavolcanic protolith and led to BIF precipitation. This structural arrangement is consistent with the remote sensing data, which revealed NNW-SSE elongated zones enriched in iron that correspond to the BIF bands identified in the field.

### Petrography and mineralogy

**Fig. 8:**
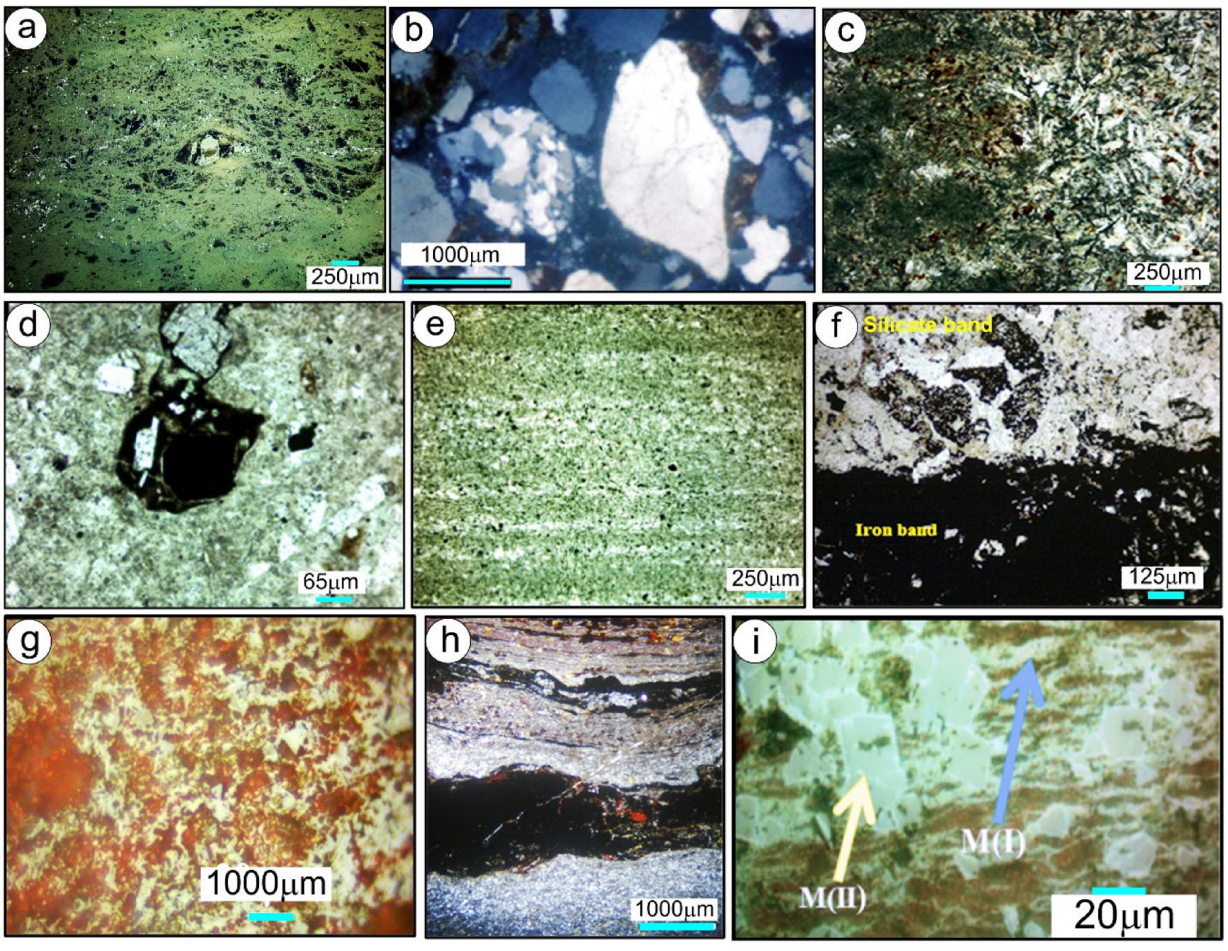
(**a**) Photomicrograph of serpentinite showing fine carbonate crystals intergrown with talc flakes and fibrous antigorite. Sheared chromite crystals and disseminated magnetite are also present (RL.), (**b**) Photomicrograph (C.N.) showing a quartz-carbonate assemblage consisting of both polycrystalline and monocrystalline quartz, with remnants of carbonate cleavage features embedded in a fine-grained carbonate matrix, (**c**) Photomicrograph showing hemi-crystalline metabasalt is grading from fine-grained basalt composed of laths of plagioclase in intersertal glass groundmass. (C.N.), (**d**) Photomicrograph of mylonite composed of lithic and crystal fragments, chlorite and epidote are replaced by magnetite, which may replace the rock fragment as a whole (PPL), (**e**) Ultramylonite composed of fine-crushed bands and ultrafine-crushed ones; reflected light, under oil immersion (**f**) Martitized magnetite–silicate facies are composed of alternating bands of iron oxide-rich and silicate-rich material (PPL.), (**g**) Iron-rich mesobands consist of ultrafine (~ 3 μm) anhedral to subhedral magnetite crystals that have undergone complete alteration to platy hematite, (**h**) Composition of magnetite-chert-jasper facies meso-banding (C.N.), (**i**) Porphyroblasts martitized magnetite cross cutting fine-grained martitized magnetite micro-band, (reflected light; under oil immersion).

Petrographic analysis of thin sections reveals that the serpentinite is predominantly composed of irregular patches of antigorite, intimately mixed with carbonate minerals and talc (Fig. 8a). The most common opaque mineral is chromite, which occurs as subhedral, deformed crystals reaching up to 3 mm in length and 0.9 mm in width. These chromite crystals show varying degrees of mechanical fragmentation and are commonly aligned into linear aggregates due to intense shearing. In some instances, magnetite partially replaces chromite along closely spaced, parallel shear planes. The talc-carbonate sheets are chiefly composed of anhedral to subhedral magnesite crystals or patches, intergrown with wavy talc flakes that often contain relict antigorite. Opaque phases include chromite, disseminated magnetite, and minor pyrite. The quartz-carbonate rocks are dominated by quartz grains embedded in a carbonate matrix. Opaque minerals in these rocks are primarily iron oxides and pyrite (Fig. 8b).

The massive metavolcanic rocks exposed along the margins of the Fatira Shear Zone are primarily of elemental composition, including metadolerite and metabasalt. Metadolerite is a massive, greenish-colored rock that is fine- to medium-grained, composed mainly of phenocrysts of plagioclase and augite set in a groundmass of altered plagioclase, displaying porphyritic, ophitic, and diabasic textures. Secondary minerals include hornblende, actinolite, actinolitic hornblende, occasional augite, chlorite, epidote, calcite, and opaque oxides.

Metabasalt is a massive, greyish-green rock that is fine-grained to glassy, composed mainly of phenocrysts of plagioclase and altered pyroxene set in a finer-grained to glassy groundmass of plagioclase laths, displaying porphyritic, intersertal, and variolitic textures (Fig. 8c). Secondary minerals include chlorite, calcite, actinolite, sericite, epidote, zoisite, quartz, and iron oxides.

The sheared metavolcanics exhibit a range of textures and mineralogical compositions depending on the degree of shearing. Protomylonite is variably foliated and ranges in colour from greenish grey to dark green, composed of lithic fragments of metadolerite and/or metabasalt (Fig. 8d) set in a fine crushed matrix partly or entirely replaced by magnetite. Mylonite rocks are banded, foliated, and greyish green, consisting of deformed rock and crystal fragments set in a foliated, finely crushed matrix with a porphyroclastic texture. Ultramylonite rocks are the most common type among the sheared metavolcanics, characterized by alternated light (fine-grained) and dark (ultrafine-grained) bands in ribbon-shaped patterns at centimetre and millimetre scales (Fig. 8e). The light bands are composed of crushed crystal fragments and shards and variable amounts of epidote, calcite, quartz, albite, and disseminated opaque oxides. In contrast, the dark bands consist of powder-sized components of crushed rock, with variable amounts of chlorite, actinolite, and epidote, and transition into banded chert.

### Mineralogy of BIF

Banded iron formations in the Fatira area are hosted in the intensively sheared parts of the metavolcanics, primarily within mylonite and ultramylonite rocks. BIFs are distinctly distributed in these sheared rocks, occurring as single or multiple bands (Fig. 8f–h).

The single bands are found at the outer peripheries of the shear zone (mylonite rocks). They are relatively thick (up to 4 m), exhibit gradational contacts with the host rocks, and contain significant remnants (up to 1 m) of the original sheared metavolcanic rocks (Fig. 2f). The single bands or lenses are generally brownish. The martitized magnetite-silicate facies represent them.

Martitized magnetite-silicate facies are composed of alternating bands of iron oxide-rich and silicate-rich material (Fig. 8f). Silicate-rich mesobands are composed mainly of rock fragments and shards embedded in a matrix of finer-grained material of the same rock composition. Iron-rich mesobands consist primarily of opaque iron oxides with small relict islands and remnants of altered sheared mylonitic rock fragments; the latter exhibit notable concave-convex relationships with the iron oxides. This observation may indicate that iron-rich bands form at the expense of fine silicate-rich bands. Under the reflected light microscope, the iron-rich mesobands comprise ultrafine grains, about 3 μm, of anhedral to subhedral magnetite crystals completely altered into platy hematite (martite; Fig. 8g). Relatively larger crystals, up to 10 μm across, can be observed; they are partially martitized along their outer peripheries and crystallization planes.

The multiple-occurring BIF bands are found in the strongly sheared parts of sheeted dykes in the central part of the Fatira Shear Zone, where the metavolcanic sheeted dykes are affected by intense shearing, losing their inter-boundaries and transforming mainly into ultramylonite. The contacts of the multiple-occurring bands are sharp and separated by thin ultramylonite bands with small remnants inside the iron bands (Fig. 8h). Multiple-occurring BIF bands show a direct relationship with dolerite dykes, which surround the BIF exposures from the north and south. The multiple-occurring BIFs are generally hard, prominent bands with red, brown, black, and grey colours, composed of magnetite-chert-jasper facies. The magnetite-chert-jasper facies consists of iron oxide-rich mesobands alternating with chert-jasper and calcite mesobands (Fig. 8h). The chert-jasper meso-bands are up to 2 cm thick, blood red, and mainly composed of fine-grained quartz, hematite dust with lensoidal relics of silicified altered mylonite of variable sizes aligned parallel to the general BIF banding. The individual chert mesoband consists of alternating fine-grained and very fine-grained quartz micro-bands. The iron mesobands are up to 3 cm thick and primarily composed of magnetite, containing less silicified altered mylonite relics. Chert micro-bands dissect these bands parallel to the general banding. The iron-rich microband consists of fine-grained martitized magnetite crosscut by euhedral to subhedral porphyroblasts, with martitization and randomly orientated magnetite up to 70 μm across (Fig. 8i).

### Geochemistry

#### Metavolcanic host rocks

It is essential to acknowledge that the Fatira BIFs and their associated rocks have undergone substantial post-depositional alteration, including regional metamorphism and tectono-hydrothermal modification. These processes may have partially obscured the primary geochemical signature, especially for mobile elements. So, it’s necessary to be cautious when using regular geochemical discrimination diagrams. In this study, interpretations are predominantly based on relatively stable elements, including High Field Strength Elements (HFSEs) and Rare Earth Elements (REEs), which are typically more resistant to alteration. Additionally, geochemical results are combined with petrographic observations and structural data to differentiate primary depositional features from secondary hydrothermal overprints. This integrated approach helps lower the chance of confusing alteration-induced signatures with primary geochemical signals.

**Fig. 9:**
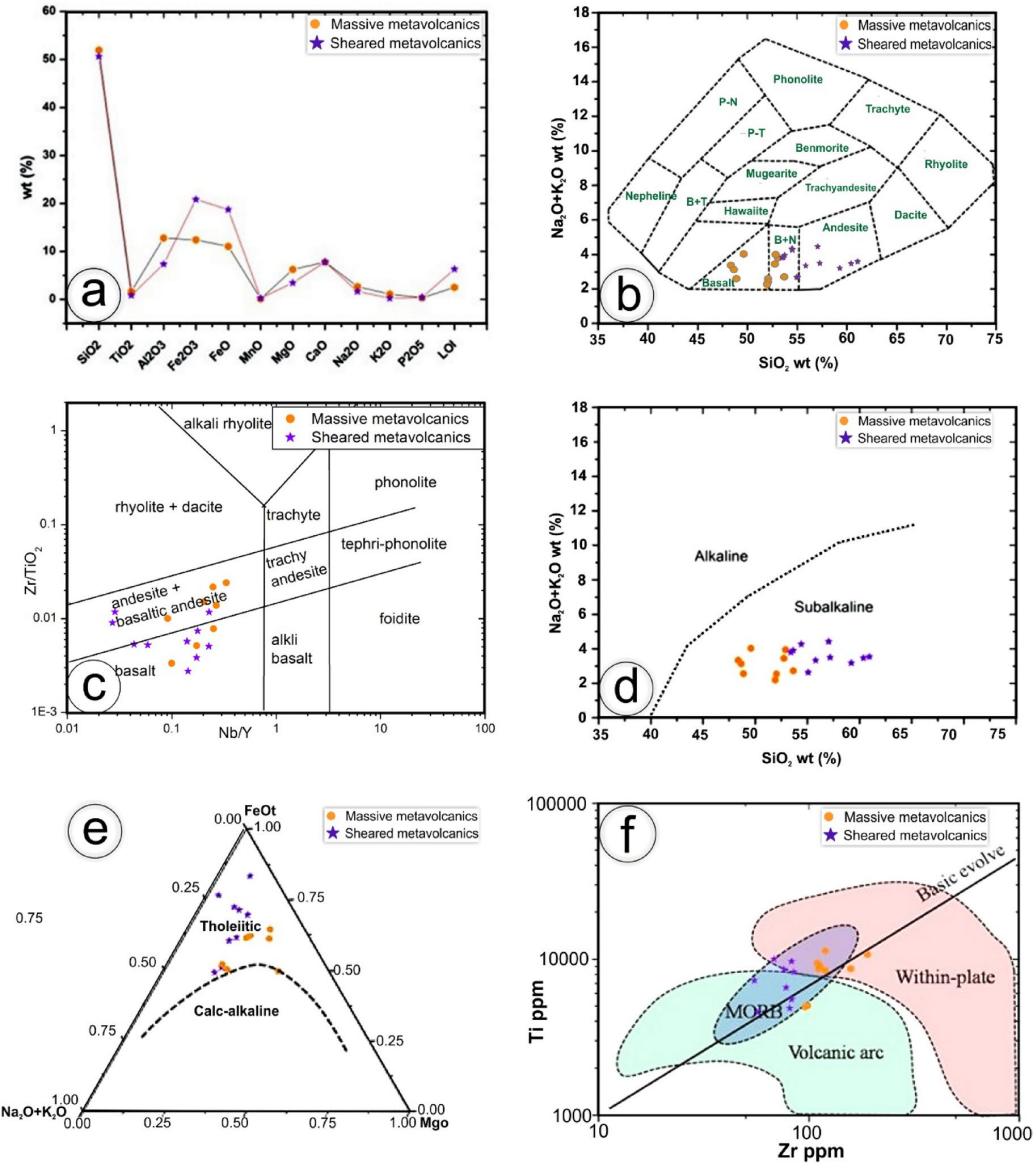
(**a**) Spider diagram of average major composition of massive metavolcanics and sheared metavolcanics (mylonite and ultramylonites), (**b**) Plot metavolcanics on SiO_2_ vs. (Na_2_O+K_2_O) diagram of Cox^65^, (**c**) Plot metavolcanics on the Alkalis-silica diagram of Irvine and Baragar^67^, (**d**) Plot metavolcanics on alkali-silica diagram of Irvine and Baragar^67^, (**e**) Plot metavolcanics on the AFM diagram of Irvine and Baragar^67^, (**f**) Plot metavolcanic on Zr vs. Ti diagram of Pearce^66^, MORB = Mid-oceanic ridge basalts.

The geochemical compositions of massive metavolcanics, sheared metavolcanics, Fatira banded iron formation, and banded chert are presented in Tables 4, 5, 6, and 7. The massive metavolcanics and sheared metavolcanics show significant pattern similarity for most major element components (Fig. 9a); however, the sheared rocks are relatively depleted in most major elements except for iron. According to the Na_2_O + K_2_O versus SiO_2_ binary diagram of^65^, the massive metavolcanic samples lie in the basalt field, with three samples in the basaltic andesite field. In contrast, the sheared metavolcanic samples fall into the andesite category, with two samples in the basaltic andesite field (Fig. 9b). This gradation from basalt to andesite rocks parallels the transition from massive to sheared rocks, possibly due to the increasing Si content associated with the silicification of the rocks during shearing events. The Zr/TiO₂ versus Nb/Y classification diagram (Fig. 9c, Pearce^66^) reveals that metavolcanic rocks are predominantly basaltic to andesitic, with minimal geochemical variation between massive and sheared units.

**Table 4:** Major oxide composition trace element and Rare earth element (REE) contents of massive metavolcanic rocks.

	1	2	3	4	5	6	7	8	9	10
SiO_2_	50.96	52.04	48.66	48.6	49.59	52.9	53.42	53.65	52.75	53.42
TiO_2_	0.14	0.67	0.76	3.65	1.51	1.55	1.57	0.81	1.9	1.57
Al_2_O_3_	3.44	7.8	11.45	12.3	11.47	12.35	14.77	11	14.72	14.77
Fe_2_O_3_	16.54	25.2	13.36	15.4	14.24	9.15	11.25	14.84	12.05	11.25
FeO	14.88	22.67	12.02	13.87	12.8	8.23	10.12	13.35	10.84	10.12
MnO	0.32	0.17	0.2	0.04	0.17	0.03	0.11	0.24	0.02	0.11
MgO	3.91	1.93	4.31	5.7	9.08	7.25	6.2	4.3	6.6	6.2
CaO	11.76	5.43	16.33	7.25	6.61	7.75	7.18	7	5.15	7.18
Na_2_O	0.58	1.97	2.25	2.25	2.34	2.85	2.54	2.34	2.65	2.54
K_2_O	0.03	0.57	0.89	1.05	1.69	1.1	1.26	0.39	0.8	1.26
P_2_O_5_	0.16	0.99	0.07	0.9	0.34	0.4	0.3	0.07	0.3	0.3
LOI	11.92	2.92	1.41	2.5	2.66	4.5	0.1	5.06	2.8	0.1
Ba	765	405	700	398	339	245	557	316	420	
Rb	42	28	35	30	23	27	30	14	15	
Sr	538	606	496	587	661	982	823	215	305	
Y	5	8	2	12	10	11	19	27.4	7	
Zr	111	113	110	47	26	22	158	81	57	
Nb	6	6	6	6	9	8	15	2.5	n.d	
Th	1	2	3	3	3	3	2	n.d	n.d	
Pb	14	6	11	10	6	3	2	12	10	
Zn	80	88	92	102	120	105	2	98	62	
Cu	36	43	27	23	15	11	2	45	90	
Ni	106	152	122	87	70	104	111	20	9	
V	215	244	217	416	680	361	144	152	165	
Cr	254	287	175	295	147	354	312	71	41	
Co	58	66	61	52	55	40	2	57	45	
Sn	5	2	5	2	2	2	2	n.d	n.d	
La	6.4	5.6	8.1							
Ce	16	14	19							
Pr	6.9	6.5	11							
Nd	11.8	12.2	13							
Sm	0.9	0.6	1							
Eu	1	1.1	1							
Gd	10	13	10							
Tb	1.7	2.3	3							
Dy	5.2	5.8	4.2							
Ho	0.1	0.1	0.1							
Er	4.6	4.9	4							
Yb	3.9	3.8	3							
Lu	1.4	1.7	2							

**Table 5:** Major oxide composition trace element and Rare earth element (REE) contents of Sheared metavolcanic rocks.

	1	2	3	4	5	6	7	8	9	10
SiO_2_	59.95	55.79	57.19	53.64	54.35	60.38	59.22	55.08	60.95	59.95
TiO_2_	1.79	1.41	1.88	1.45	1.45	0.92	0.82	1.43	0.84	1.79
Al_2_O_3_	14.18	10.98	11.89	12.27	14.65	10.59	13.98	10.25	10.48	14.18
Fe_2_O_3_	8.89	16.64	12.52	13.24	8.15	9.02	10.95	17.37	10.16	8.89
FeO	7.99	14.97	11.27	11.9	7.33	8.12	9.85	15.63	9.14	7.99
MnO	0.13	0.26	0.18	0.14	0.11	0.15	0.17	0.29	0.15	0.13
MgO	2.52	3.16	3.58	7.44	5.3	1.68	2.8	3.6	1.52	2.52
CaO	3.42	7.45	5.48	6.2	6.55	4.79	6.35	5.9	7.02	3.42
Na_2_O	4.6	2.31	3.23	2.71	3.9	1.57	2.25	2.16	2.08	4.6
K_2_O	0.97	1.02	0.27	1.2	1.45	1.9	0.93	0.47	1.46	0.97
P_2_O_5_	0.35	0.17	0.22	0.31	0.3	0.15	0.09	0.16	0.08	0.35
LOI	2.89	0.52	2.55	0.1	3.5	6.97	1.13	3	4.25	2.89
Ba	201	201	70	172	301	611	565	339	637	70
Rb	8	8	9	16	10	57	31	12	34	9
Sr	263	263	72	117	332	183	474	275	136	72
Y	20	20	9	7	17	37	22	23	35	9
Zr	120	120	57	40	76	83	96	76	99	57
Nb	9	9	9	n.d	n.d	1	5	n.d	n.d	9
Th	2	2	2	n.d	n.d	1	2	n.d	n.d	2
Pb	6	6	10	10	10	7	7	7	7	10
Zn	78	78	44	65	92	104	63	103	79	44
Cu	21	21	4	72	112	33	31	45	57	4
Ni	2	2	2	1.8	2	1	3	1.8	9	2
V	269	269	50	126	351	134	152	214	117	50
Cr	2	2	3	18	15	5	14	4	15	3
Co	51	51	2	19	55	38	35	44	44	2
Sn	5	5	2	n.d	n.d	1	4	n.d	n.d	2
La	10	12	12							
Ce	27	30	28							
Pr	7	10	11							
Nd	16	20	20							
Sm	1.5	2	1							
Eu	1	1.3	1.2							
Gd	10	12	12							
Tb	2	3	3							
Dy	4	4.8	3							
Ho	0.1	0.1	0.1							
Er	5	7	5							
Yb	3	3	2							
Lu	2	2	2							

**Table 6:** Major oxide composition trace element and Rare earth element (REE) contents of Fatira banded iron formation.

	1	2	3	4	5	6	7	8	9	10
SiO_2_	24.3	23.1	25.08	22.27	24.02	20.89	23.6	27.88	26.1	30.5
TiO_2_	0.12	0.13	0.19	0.15	0.1	0.21	0.15	0.25	0.27	0.13
Al_2_O_3_	1.2	1.1	2.27	1.69	1.1	1.33	1.14	2.3	2.41	1.38
FeO	0.07	0.07	1.02	1.14	1.01	1.18	0.51	2.32	2.2	0.78
Fe_2_O_3_	65.84	66.34	61.26	67.61	69.5	67.32	66.74	51.85	52.15	50.49
MnO	0.06	0.07	0.08	0.07	0.07	0.09	0.08	0.11	0.14	0.14
MgO	0.01	0.02	0.07	0.01	0.01	0.01	0.01	0.28	0.25	0.01
CaO	5.03	5.8	5.78	4.39	3.27	5.8	4.63	9.27	10.3	10.95
Na_2_O	0.81	0.71	0.66	0.8	0.81	0.79	0.75	0.71	0.68	0.75
K_2_O	0.06	0.05	0.5	0.44	0.07	0.01	0.04	0.54	0.6	0.01
P_2_O_5_	2.21	2.31	3.06	1.85	0.74	2.6	1.94	1.52	1.61	2.71
LOI	0.2	0.2	0.74	0.66	0.23	0.92	0.79	3.44	3.34	2.84
Ba	25	25	27	36	36	35	14	85	84	86
Rb	11	12	12	25	10	10	10	12	13	8
Sr	12	13	20	45	14	13	12	25	26	59
Y	28.4	27.4	8	12	1.8	7	5	4	5	1.8
Zr	17	18	20	22	17	17	18	22	24	23
Nb	n.d	n.d	6	n.d	n.d	n.d	n.d	n.d	n.d	n.d
Th	n.d	n.d	2	n.d	n.d	n.d	n.d	n.d	n.d	n.d
Pb	20	21	29	18	14	20	15	16	18	10
Zn	5	6	11	5	3	8	5	13	12	7
Cu	n.d	n.d	2	9	3	5	3	13	12	n.d
Ni	4	4	3	4	4	3	3	3	3	2.5
V	27	25	50	88	27	37	30	47	45	17
Cr	9	9	21	7	7	6	10	29	28	29
Co	40	41	23	39	38	40	32	14	14	13
Sn	n.d	n.d	2	n.d	n.d	n.d	n.d	n.d	n.d	n.d
La	6	7.7	5.2							
Ce	0.2	0.1	0.1							
Pr	29	30.6	32							
Nd	20	29.5	18							
Sm	0.11	0.1	0.1							
Eu	0.2	1	0.1							
Gd	45	70	43							
Tb	7	9.3	6							
Dy	0.3	1.3	0.1							
Ho	0.4	1.3	0.1							
Er	0.5	1.7	0.1							
Yb	4	7.4	3.2							
Lu	8	8.7	6							

**Table 7:** Major oxide composition trace element and Rare earth element (REE) contents of banded chert.

	1	2	3	4	5
SiO_2_	52.37	45.03	57.41	39.57	64.5
TiO_2_	0.18	0.56	0.08	0.08	0.87
Al_2_O_3_	2.32	5.62	0.9	2.47	12.43
FeO	3.38	7.1	n.d	n.d	n.d
Fe_2_O_3_	25.82	33.43	24.61	9.94	10.85
MnO	0.12	0.16	0.06	0.22	0.15
MgO	0.56	1.62	0.13	0.52	3.11
CaO	4.45	6.47	8.7	30.72	1.86
Na_2_O	0.65	1.05	0.65	1.02	3.9
K_2_O	0.01	0.73	0.24	0.21	0.02
P_2_O_5_	0.1	0.37	6.37	0.13	0.08
LOI	13.28	4.66	1.09	15.11	1.93
Ba	102	212	20	62	192
Rb	8	17	10	8	7
Sr	36	36	118	204	242
Y	2	16	24	2	22
Zr	56	45	31	37	106
Nb	n.d	n.d	7	4	10
Th	n.d	n.d	2	2	15
Pb	18	6	25	4	9
Zn	35	35	7	19	103
Cu	5	39	7	12	46
Ni	1.8	1.8	2	2	4
V	81	83	23	14	129
Cr	7	15	11	2	7
Co	3	4	2	16	41
Sn	n.d	n.d	1	3	6
La	15	10			
Ce	21	27			
Pr	22	7			
Nd	23	16			
Sm	1	1.5			
Eu	1	1			
Gd	28	10			
Tb	5	2			
Dy	4.8	4			
Ho	0.1	0.1			
Er	4	5			
Yb	5	3			
Lu	4	2			

**Fig. 10:**
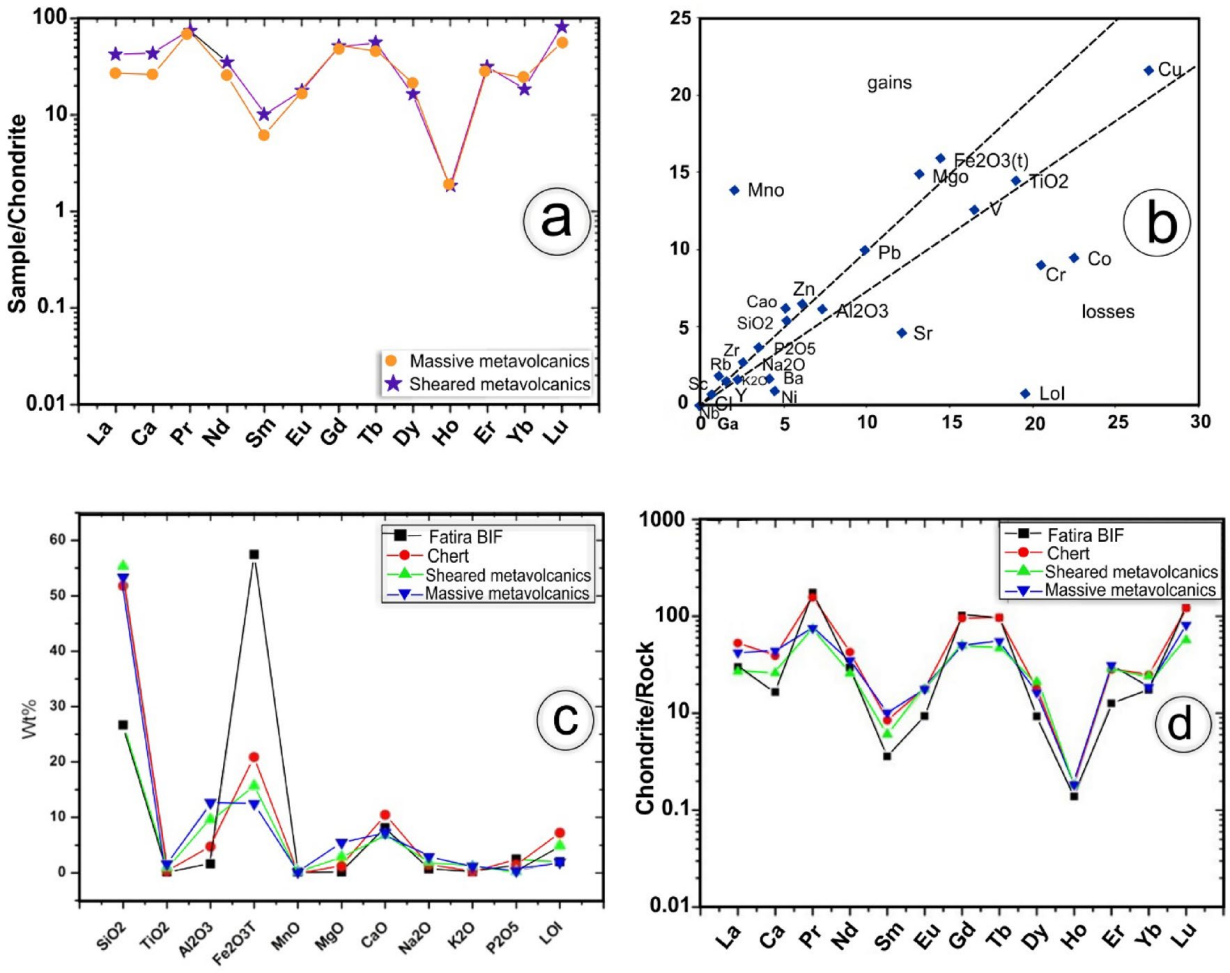
(**a**) Chondrite-normalized REE patterns (Sun and McDonough)^68^ of massive metavolcanics and sheared metavolcanics (mylonite and ultramylonites) rocks, (**b**) Isocon analysis^69^ performed using the least-mobile elements Zr and Y, (**c**) Spider diagram of average major oxides of Fatira BIF compared with banded chert, sheared metavolcanic rocks (mylonites and ultramylonites) and massive metavolcanics; (**d**) Chondrite-normalized REE patterns (Sun and McDonough)^68^ of Fatira BIF, banded chert, massive metavolcanics and sheared metavolcanics (mylonite and ultramylonites) rocks.

Based on the alkali-silica diagram^67^ (Fig. 9d), the metavolcanic rocks exhibit subalkaline characteristics. According to the AFM diagram of Irvine and Baragar^67^, most samples display a tholeiitic nature (Fig. 9e). Using the Zr vs. Ti diagram after Pearce^66^, the metavolcanic rocks fall within the MORB field (Fig. 9f). The chondrite-normalized REE patterns for the metavolcanic rocks (Fig. 10a) exhibit smooth convex (M-type) or concave (W-type) shapes^67^, suggesting an increasing degree of water-rock interaction during hydrothermal alteration processes.

To quantify mass transfer during hydrothermal alteration, isocon analysis^69^ was performed using the least-mobile elements Zr and Y as the reference immobile set. The slope of the isocon (k = 0.73) defines the relative mass change between the altered and unaltered metavolcanics (Fig. 10b). The overall mass change implied by this slope was + 37%, indicating a net mass gain in the altered sample, consistent with hydrothermal addition and oxide enrichment.

Elements plotting above the isocon indicate net addition during alteration, including MnO (+ 62%), Fe₂O₃(t) (+ 51%), MgO (+ 55%), CaO (+ 65%), K₂O (+ 106%), and P₂O₅ (+ 42%), consistent with addition from Fe–Mg–Ca–K-bearing hydrothermal fluids. In contrast, elements plotting below the isocon, such as LOI (− 95%), Sr (− 47%), Ni (− 73%), Cr (− 40%), and Co (− 42%), indicate depletion, consistent with leaching of volatiles and loss of mobile transition metals during fluid–rock interaction. The behavior of Zr (− 3.5%) and Y (+ 37%) supports their use as immobile reference elements.

The percent mass change for each element (Δ%) was calculated using the relationship:

$$\:\varDelta\:\%=\frac{{\mathrm{C}}_{\mathrm{a}}}{\mathrm{K}.{\mathrm{C}}_{\mathrm{u}}}-1\times\:100$$

Where (C_a_) and (C_u_) represent altered and unaltered concentrations, respectively, and k is the isocon slope.

A sensitivity test using alternative immobile element combinations produced consistent results:

- Set A (Ti + Zr) yielded k = 0.73 and Δ_mass_ = + 37%,
- Set B (Zr + Y) yielded k = 0.73 and Δ_mass_ = + 37%,
- Set C (Ti + Y) yielded k = 0.88 and Δ_mass_ = + 14%,
- Set D (Ti + Zr + Y) yielded k = 0.82 and Δ_mass_ = + 22%.

These minor variations (calculated as average concentration ratios) confirm the robustness of the mass balance interpretation, showing that the hydrothermal alteration of metavolcanics was characterized by Fe–Mg–Ca–K enrichment and volatile–Sr–Ni depletion, consistent with propylitic to sericitic alteration zones.

#### Banded iron formation

Major element compositions of the Fatira banded iron formation show that Fe_2_O_3_ and SiO_2_ are the main components. The Fe_2_O_3_ content ranges from 38.77 to 69.5 wt%, averaging 57.47 wt%. In comparison, the SiO_2_ content ranges from 20.89 to 36.03 wt% with an average of 26.67 wt% (Fig. 10c). The average chemical composition of the Fatira BIF is compared with that of banded chert, massive metavolcanics, and sheared metavolcanics (mylonites and ultramylonites) (Fig. 10d). It has been observed that they exhibit the same pattern, with a relative decrease in most major oxides from massive metavolcanics through sheared metavolcanics and chert to BIF, except for Fe_2_O_3_. The chondrite-normalized REE patterns of the Banded Iron Formation and their corresponding host rocks, which consist of massive/sheared metavolcanics and bedded chert, are depicted in Fig. 10d. This pattern reveals a convex (M-type) or concave (W-type) shape^70^. It is suggested that fluid-rock interactions in this context result in the REE tetrad effect, which is linked to hydrothermal vent processes.

## Discussions

### Lithological and iron mapping

As mentioned, remote sensing is an effective tool for lithological and structural mapping. The tonal variations in the color composites played an essential and effective role in discriminating among the different rock units, aiding remapping and improving the geological map of the study area. High spatial resolution of FCC 432-RGB from ASTER data was a vital tools for the monitoring of the different valleys in the mapped area as well as it successfully demarcates between the lithological units (Fig. 3a). It was easy to discriminate and differentiate through the generated color imageries (Fig. 3) between the massive metavolcanics and sheared metavolcanics which are considered the domain source of the BIF in our area. Decorrelated FCC 765-RGB (Fig. 3b) proved its effectiveness through its ability to distinguish between the entire rock units. Moreover, the other band ratios (Fig. 3c,d) enable us to differentiate between the Dokhan volcanics, molasse-type sediments, and the syn- and late-granites. The field data have verified these discriminations of the lithological units and indicate that the selected enhanced and transformed images (Fig. 3) accurately highlight the lithological contacts.

The allocation and mapping of iron oxides have been conducted depending on the utilization of a set of grey-scaled band ratios of Landsat-8, like 6/2, 6/4, 6/5, and PC3 (Fig. 4a–d), which have been examined and proved their validity in the detection of different iron oxides around the world, 49, 50, 71, particularly within the Egyptian Eastern Desert^19,71–73^. Utilizing the SAM technique applied to ASTER data revealed the spatial distribution of the four common iron minerals, including hematite, magnetite, jarosite, and goethite (Fig. 5a–d). The spatial distribution and sites of the iron oxides, as determined from both Landsat-8 and ASTER, coincided across most of the investigated area (Fig. 4a–d and 5a–d). The correlated sites form the propylitic zones from ASTER, CO3-OH bearing minerals as alteration zones, along with the spatial distribution of iron by Landsat-8 and ASTER, revealed that there is a coincidence or overlap between these alteration zones and the detected iron sites, which display the role of the alteration processes in the increase of the iron in the presented area. So, hydrothermal fluids are considered one of the primary iron sources in the presented area, primarily through the metavolcanics.

**Fig. 11:**
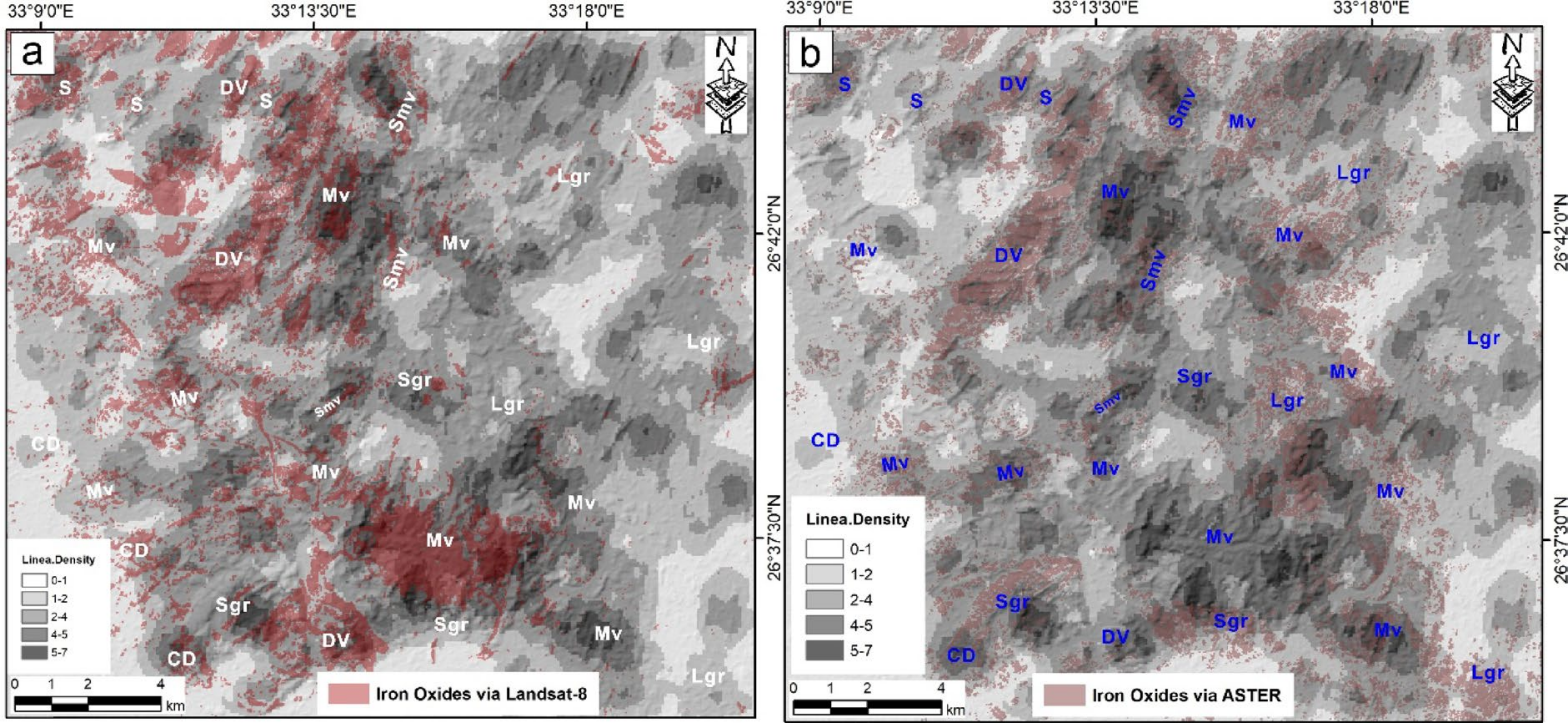
Spatial distribution of iron oxides vs. the spatial distribution of lineament density; (**a**) By Landsat-8, (**b**) By ASTER. This figure was created and processed by ENVI v. 5.6.2. software: https://www.l3harrisgeospatial.com/Software-Technology/ENVI), mainly used for image processing, and ArcGIS Desktop 10.8. (https://www.esri.com/en-us/arcgis/products/arcgis-desktop/overview/).

The integration of lineament density and surface distribution of iron oxides, either from Landsat-8 or ASTER, revealed that the detected iron was recorded and overlapped in areas with moderate to high lineament density and marked by metavolcanic composition (Fig. 11a,b). These overlapping of the irons with moderate to high lineament density displayed that the linear structures (e.g., fractures, joints and faults) in the area worked as conduits for the hydrothermal solution enriched by iron to pass through them and precipitate these iron oxides within the metavolcanics and other parts of the syn and late-granitic rocks which are characterized by moderate to high lineament density according to the density map Fig. 6b. So, the linear structures with domain trends such as NNE, NE, and NW, which are mostly related to the NW-Najd fault system, played an essential role in the distribution and concentration of iron oxides or BIFs in the presented area. Excluding all the grey-scale images that display the distribution of the iron in the study area, Fig. 5a of the hematite minerals shows that the Dokhan volcanics have a high concentration of hematite. This notice is due to the upper parts of the Dokhan volcanics being covered by molasse-type sediments characterized by high hematite content.

Spectral analysis revealed the presence of iron oxides across a diversity of lithological units within the study area. The present investigation, however, deliberately prioritized units exhibiting a conjunctive occurrence of high iron oxide concentrations and elevated lineament density. This paragenesis of geochemical and structural attributes is interpreted as diagnostic of sheared and massive volcanic sequences. Although ferruginous signatures were also identified within other lithologies, including syn-tectonic granitoids and Cretaceous deposits, the Dokhan volcanics were systematically excluded as a potential primary source. This methodological exclusion is predicated on the unit’s extensive stratigraphic obscuration by a molasse sedimentary cover. This overlying sequence is posited to be significantly enriched in ferruginous constituents, derived from the erosional dismantling of pre-existing volcanic terrains. Consequently, the anomalously high iron oxide signatures recorded over the Dokhan volcanics are not interpreted as being representative of the underlying bedrock. Still, they are more parsimoniously explained as a supergene expression arising from the ferruginous molasse cover.

### Field and laboratory validation

Integrating field observations and laboratory analyses has been critical in validating the remote sensing data and providing a deeper understanding of the Banded Iron Formations (BIFs) in the Wadi Fatira area. Field investigations revealed that the BIFs are spatially and genetically associated with the Fatira Shear Zone, which was pivotal in their localization. The shear zone, characterized by intense deformation, hosts the BIFs in two distinct settings: the outer peripheries, where single, thick bands occur within mylonite rocks, and the central part, where multiple, thinner bands are intercalated with ultramylonite layers. The gradational contacts between the BIF bands and the host rocks in the outer shear zone suggest a gradual replacement process, while the sharp contacts and silicified mylonite relics in the central shear zone point to intense fluid-rock interaction. The association of these bands with dolerite dykes further supports the role of hydrothermal activity in their formation. Field observations also highlighted the structural control of the shear zone on BIF distribution, with NE-SW-trending shear zones acting as conduits for hydrothermal fluids that altered the metavolcanic protolith and precipitated the BIFs. This structural framework aligns with the remote sensing data, which identified NNW-SSE elongated Fe-enriched zones corresponding to the BIF bands observed in the field.

Laboratory investigations provided vital insights into the mineralogical and geochemical features of the BIFs and their host rocks, illuminating their formation processes. The BIFs show distinct mineralogical facies that indicate different levels of hydrothermal alteration. In the outer shear zone, the martitized magnetite-silicate facies consists of alternating bands of iron oxides (mainly martitized magnetite) and silicate-rich material, signalling oxidative alteration conditions. Conversely, the central shear zone contains magnetite-chert-jasper facies, characterised by alternating bands of magnetite, chert, and jasper, with silicified mylonite relics and porphyroblasts of magnetite and hematite, suggesting recrystallization under hydrothermal conditions. Geochemical analyses demonstrated that Fe2O3 and SiO2, with minor amounts of Al2O3, CaO, MgO, and P2O5, dominate the BIFs. Meanwhile, the sheared metavolcanic rocks show a gradual depletion in major elements (except Fe2O3) from massive metavolcanics to ultramylonites, consistent with the leaching of mafic components by hydrothermal fluids. The chondrite-normalised REE patterns of the BIFs exhibit convex (M-type) or concave (W-type) shapes, indicative of fluid-rock interaction during hydrothermal alteration.

**Fig. 12:**
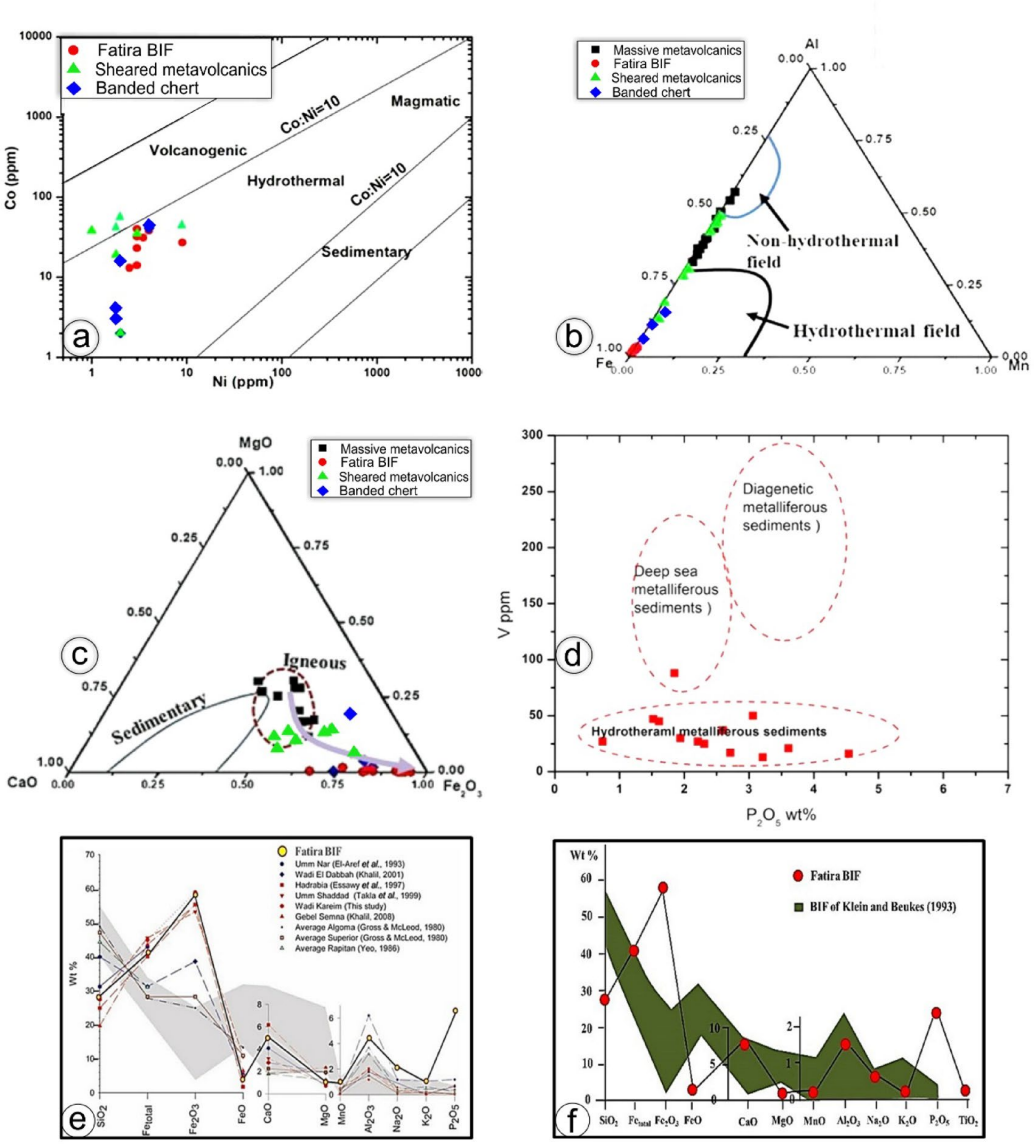
(**a**) Plotting of the BIF, chert, and the sheared metavolcanics (mylonites and ultramylonites) samples on the Ni-Co binary diagram of Bajwah et al.^74^, (**b**) The plotted BIF samples and their host rocks on the Fe-Mn-Al ternary diagram after Adachi et al.^75^, (**c**) The plotted BIF samples and their host rocks on the “Fe2O3-Mgo-CaO” ternary diagram after Moine and De La Roche^76^, (**d**) Plotting of BIF samples on the Y versus P_2_O_5_ binary diagram of Marchig et al.^77^, (**e**) Average major composition of Fatira BIF compared to averages Algoma and Superior type BIFs (shaded green) from Klein and Beukes^78^, (**f**) Averages major element composition of Fatira BIF compared of known Egyptian BIFs Khalil and El-Shazly^79^, Algoma, Superior, and Rapitan type BIFs from Klein^6^,the shaded area represents range for Algoma and Superior type BIFs^6^.

Geochemical plots, such as the Ni vs. Co shows the distribution of data points suggests that Fatira BIF and banded chert samples fall within the hydrothermal field, while sheared and massive metavolcanics are more scattered, indicating a mix of volcanic and hydrothermal influences (Fig. 12a). In the (Al-Fe-Mn) ternary diagram the placement of data points suggests that Fatira BIF and banded chert samples are primarily situated in the hydrothermal zone, while sheared and massive metavolcanics show a broader distribution, hinting at a combination of volcanic and hydrothermal effects (Fig. 12b). The Fe_2_O_3_-MgO-CaO ternary diagram (Fig. 12c), reveals that Fatira BIF samples, concentrated near the Fe_2_O_3_ apex, while banded chert, sheared metavolcanics, and massive metavolcanics show a transition from igneous to the Fe_2_O_3_apex reflecting the hydrothermally influences. Figure 12d shows that the BIF (Banded Iron Formation) samples are situated in the hydrothermal/metalliferous sediments field with V concentrations of 50 to 150 ppm and P_2_O_5_ content of 1 to 4 wt%, indicating a strong hydrothermal influence with moderate vanadium enrichment and low to moderate phosphorus levels.

The Fatira Shear Zone emerges as a critical factor in the localization of BIFs, acting as a pathway for hydrothermal fluids that altered the metavolcanic protolith and precipitated the iron-rich bands. The hydrothermal processes, facilitated by shear-zone dynamics, are further supported by mineralogical and geochemical evidence, highlighting the complex interplay among structural, hydrothermal, and geochemical factors in forming the Fatira BIFs.

### Formation mechanisms and hydrothermal processes

The formation of the Fatira BIFs is closely linked to hydrothermal processes facilitated by the Fatira Shear Zone. The geochemical and mineralogical data provide compelling evidence for this model. The geochemical plots, including the Ni-Co binary diagram of Bajwah et al.^74^, Fe-Mn-Al ternary diagram after Adachi et al.^75^, Fe_2_O_3_-MgO-CaO ternary diagram after Moine and De La Roche^76^, and Y versus P_2_O_5_ binary diagram of Marchig et al.^77^, demonstrate a clear transition from non-hydrothermal massive metavolcanics to hydrothermally altered sheared metavolcanics and, ultimately, to hydrothermal chert and BIFs (Fig. 12c,d). This gradation supports the hypothesis that hydrothermal fluids, channeled through the shear zones, altered the metavolcanic protolith and precipitated the BIFs. The increase in Fe_2_O_3_content with decreasing CaO and MgO further supports a replacement relationship, in which hydrothermal fluids leached mafic components from the host rocks and deposited iron-rich minerals.

The Fatira Shear Zone played a critical role in the localization of BIFs. The intense shearing of metavolcanic rocks created pathways for hydrothermal fluids, forming protomylonite, mylonite, and ultramylonite^53^. These sheared rocks hosted the BIFs, with single, thick bands (3–4 m) at the outer boundaries of the shear zone and multiple, thinner bands in the central, more intensely sheared regions. The mineralogical composition of the BIFs, particularly the presence of martitized magnetite and chert-jasper bands, reflects the varying degrees of fluid-rock interaction along the shear zone.

### Comparison with local and regional BIFs

The Fatira BIFs share characteristics with both Algoma- and Superior-type BIFs. The Fatira BIFs exhibit relatively elevated Fe_2_O_3_ and P_2_O_5_ contents, which reflect localized hydrothermal alteration linked to fluid-rock interaction within the Fatira Shear Zone, rather than represent fundamentally distinct BIF category Klein and Beukes^83^ (Fig. 12e). This enrichment is consistent with the hydrothermal origin of the Fatira BIFs, as supported by the geochemical plots. The Fatira BIFs also display distinct REE patterns, with convex (M-type) or concave (W-type) shapes, indicative of fluid-rock interaction during hydrothermal alteration. Compared to other Egyptian BIF deposits, the Fatira BIFs are relatively enriched in Fe_2_O_3_, CaO, Al_2_O_3_, Na_2_O, K_2_O, and P_2_O_5_, while showing similar values for Fe, SiO_2_, FeO, MgO, and MnO (Fig. 12f). These differences likely reflect variations in the formation conditions, such as the composition of the hydrothermal fluids and the nature of the protolith.

For comparison, classical low- to intermediate-grade metamorphic iron formation and weakly deformed boulders have been well described in the literature^68,78–82^. In contrast, the Fatria iron-rich rocks have been strongly modified by hydrothermal fluids along the Fatira Shear Zone, producing mineralogical and structural features that differ from those of these less-deformed examples. Based on the integrated findings, we propose a genetic model for the formation of the Fatira BIFs: (i) Protolith and shear zone formation: The metavolcanic sheeted dykes, which served as the protolith for the BIFs, were sheared along the Fatira Shear Zone, creating pathways for hydrothermal fluids; (ii) Hydrothermal alteration: Hydrothermal fluids, likely associated with dolerite dykes, altered the sheared metavolcanic rocks, leaching mafic components and depositing iron-rich minerals; (iii) BIF Precipitation: The iron-rich fluids precipitated BIFs along the shear zones, forming single thick bands at the outer boundaries and multiple thinner bands in the central, more intensely sheared regions; and (iv) Post-Formation Alteration: Subsequent hydrothermal activity further altered the BIFs, forming martitized magnetite-silicate facies and magnetite-chert-jasper facies.

## Conclusions

- The Wadi Fatira area comprises a complex of metavolcanic and sheared metavolcanic rocks, including syn-tectonic granitoids, Dokhan volcanic rocks, molasse-type sediments, and late-orogenic granitoids. Metavolcanics are of intermediate and basic composition, while sheared metavolcanic rocks include mylonite and ultramylonite types. Syn-tectonic granitoids are made up of granodiorite and tonalite, whereas the Dokhan volcanic formations are identified as rhyolite porphyry. Molasse-type sediments are recognised as polymictic conglomerates.
- A study was carried out using Landsat-8 grey scale band ratios and the SAM method to detect iron oxides in the area. The research identified zones containing chlorite and CO3-OH-bearing minerals and employed the SAM algorithm to map the spatial distribution of four common iron minerals: hematite, magnetite, jarosite, and goethite. The correlation between lineament density and the surface distribution of iron oxides suggests that the detected iron was documented and overlapped in regions characterized by moderate to high lineament density and metavolcanic composition.
- The Wadi Fatira area is part of the Barud Gneissic Complex, located along the ENE-trending dextral shear zone of the Qena-Safaga Line. The protolith of the Barud Gneissic Complex underwent crustal shortening approximately 697 Ma, leading to dextral movement along the Fatira shear zone. The Wadi Fatira area includes the Banded Iron Formations (BIFs) within extensively sheared metavolcanic sheeted dykes, shaped by the influence of the Fatira Shear Zone.
- Banded iron formations (BIFs) are found in the sheared regions of metavolcanics, mainly within mylonite and ultramylonite rocks. They show a specific distribution and can appear as single or multiple bands. They are characterized by martitized magnetite-silicate facies, consisting of alternating iron oxide-rich and silicate-rich bands. These bands are hard, prominent, and display colors such as red, brown, black, and grey; they are made up of the magnetite-chert-jasper facies.
- Remote sensing plays a vital role in lithological and structural mapping, with the FCC (Foundational Color Composite) serving as a fundamental tool. Band-ratioing and PCA improve image quality, supporting geological interpretation and mineral detection. Tonal variations within composites help distinguish different rock units, enhancing geological maps. Landsat-8’s grey-scale band ratios were utilized to map iron oxides, especially in the Egyptian Eastern Desert. The Spectral Angle Mapper technique identified the spatial distribution of common iron minerals.
- The geochemical characteristics of massive and sheared metavolcanics indicate similar trends for most significant elements, but sheared rocks show a depletion in all elements except iron. The massive metavolcanic rocks exhibit subalkaline traits and a tholeiitic nature, placing them in the MORB field.
- The formation of Fatira BIFs is linked to hydrothermal activities within the Fatira Shear Zone. Geochemical and mineralogical analyses show a progression from non-hydrothermal massive metavolcanics to hydrothermally altered sheared metavolcanics, ultimately resulting in hydrothermal chert and BIFs. Hydrothermal fluids moving through the shear zones altered the metavolcanic protolith and promoted the precipitation of BIFs. The Fatira banded iron formation contains Fe2O3 and SiO2, with Fe2O3 ranging from 38.77 to 69.5 wt% and SiO2 from 20.89 to 36.03 wt%.
- The Fatira BIFs represent a distinctive deposit of Egyptian Banded Iron Formations (BIFs), characterized by elevated levels of Fe_2_O_3_and P_2_O_5_ when compared to Algoma- and Superior-type BIFs. These formations exhibit unique Rare Earth Element (REE) patterns, suggesting a significant interaction between fluids and rock during hydrothermal alteration.
- Although remote sensing, structural analysis, and geochemical data are well integrated, some issues still need resolution. The geochemical dataset includes 15 BIF samples, 15 host metavolcanic rock samples, and 5 hydrothermal alteration samples. These samples effectively illustrate the main geochemical features of the studied lithologies, but their spatial distribution may not capture the full extent of lithological variation across the Fatira Shear Zone, particularly between its central and peripheral segments. More comprehensive, spatially distributed sampling in future research would help better constrain the range of hydrothermal alteration and enhance the genetic model of BIF formation in the study area.

## Data Availability

The datasets used and/or analyzed during the current study available from the corresponding author on reasonable request.
